# Interpretable machine learning models for predicting cognitive impairment using NHANES neuropsychological tests: nutritional and sociodemographic associations

**DOI:** 10.3389/fnut.2025.1680290

**Published:** 2026-01-14

**Authors:** Li Song, Chenlu Li, Xiaojiao Xiang, Peijia Lin

**Affiliations:** 1 Department of Neurology, The Second Affiliated Hospital of Chongqing Medical University, Chongqing, China; 2 Department of Neurology, The First Affiliated Hospital Of Chongqing Medical University, Chongqing Key Laboratory of Major Neurological and Mental Disorders, Chongqing Key Laboratory of Neurology, Chongqing, China; 3 Department of Nuclear Medicine, the Second Affiliated Hospital of Chongqing Medical University, Chongqing, China; 4 Department of Otolaryngology Head and Neck Surgery, Children’s Hospital of Chongqing Medical University, National Clinical Research Center for Children and Adolescents’ Health and Diseases, Ministry of Education Key Laboratory of Child Development and Disorders, Chongqing, China

**Keywords:** vitamin B2, cognitive impairment, nutrient synergy, NHANES, SHAP, oxidative stress, SIRT1, BDNF

## Abstract

**Background:**

Early identification of individuals at risk for cognitive impairment is essential for timely intervention and public health planning. While sociodemographic and clinical predictors are well recognized, the role of nutrition and its interactions in cognitive health remains less explored.

**Methods:**

Using data from the 2011–2014 National Health and Nutrition Examination Survey (NHANES, *n* = 2,208), we developed ensemble machine learning models (LightGBM, XGBoost, Random Forest) to predict cognitive impairment across three neuropsychological assessments (CERAD-WL, DSST, AFT). SHapley Additive exPlanations (SHAP) were applied to quantify and interpret the contribution of demographic, clinical, and nutritional predictors, as well as their interactions. To validate the nutrient interactions identified by our models, we conducted exploratory *in vitro* experiments assessing oxidative stress and neuroprotective pathways in SH-SY5Y neuronal cells.

**Results:**

Ensemble models demonstrated excellent predictive performance, consistently outperforming traditional classifiers. Key predictors included education, age, socioeconomic status, and chronic disease conditions. Among nutritional factors, vitamin B2 emerged as consistently associated with lower predicted cognitive impairment risk across all three models, with notable interactions observed with copper and vitamin E. Exploratory *in vitro* experiments supported these associations, showing reduced oxidative stress and increased expression of neuroprotective genes (SIRT1, BDNF) under vitamin B2 treatment, particularly when combined with copper or vitamin E.

**Conclusion:**

Interpretable machine learning models integrating cognitive tests with demographic, clinical, and nutritional variables can accurately predict cognitive impairment. Nutritional predictors, particularly vitamin B2 and its interactions, may contribute to model performance and biological plausibility, suggesting potential avenues for stratified monitoring strategies.

## 1 Introduction

With an aging population and increasing life expectancy, dementia has emerged as a major global health challenge, with its incidence and prevalence continuously rising (1, 2). It is estimated that by 2050, there will be more than 150 million people affected by dementia worldwide (3). Dementia is a progressive neurodegenerative disorder characterized by sequential decline in cognitive function, memory impairment, and behavioral changes, eventually leading to the loss of independent living ability (4). Therefore, early identification of dementia is crucial for timely intervention and precision treatment, which may help slow disease progression and improve patients’ quality of life (5). Current methods for early diagnosis include cognitive assessments, neuroimaging techniques, and biomarker detection (6–8). However, these approaches have notable limitations, such as low sensitivity and specificity of biomarkers, nonspecific early symptoms, and the high cost of PET scans (9–11). There is thus an urgent need for affordable, convenient, and highly specific early screening tools.

Neuropsychological testing plays a vital role in assessing cognitive function (12). These assessments are advantageous due to their low cost, ease of administration, and ability to evaluate multiple domains of cognitive decline (13). Although single tests have their limitations, combining multiple assessments with advanced statistical analyses can significantly enhance early detection and prognosis prediction of cognitive impairment (14). The CERAD Word List (CERAD-WL) test evaluates word learning and delayed recall and is highly sensitive to early dementia, but its performance may be influenced by educational and cultural background. The Digit Symbol Substitution Test (DSST) primarily measures processing speed and executive function, making it effective for identifying vascular cognitive impairment, though its specificity for dementia pathology is relatively low. The Animal Fluency Test (AFT) assesses semantic fluency, but is limited by language ability (15–17). Using these three assessments in combination enables a comprehensive evaluation of cognitive changes across multiple domains. Developing an efficient and practical large-scale screening tool by integrating multiple cognitive tests represents a current and promising strategy for early prevention of cognitive impairment.

Accumulating evidence suggests that specific nutrients may influence neurocognitive resilience in later life. Among these, vitamin B2, copper, and vitamin E have garnered particular attention due to their close involvement in neuronal survival, antioxidative capacity, and synaptic function (18–20). Taken individually, vitamin B2 (as the FAD/FMN cofactor) supports mitochondrial metabolism and the glutathione antioxidant cycle and has been linked to better cognitive performance (18); vitamin E, a membrane-phase chain-breaking antioxidant, is associated with lower dementia risk or slower functional decline (21, 22); and copper supports antioxidant enzymes such superoxide dismutase (23); while sufficient intake is necessary, excess exposure has been linked to faster cognitive decline (24, 25). Research indicates that these micronutrients often do not act in isolation but form a collaborative antioxidant network: copper-dependent enzymes initiate the clearance of superoxide anions, riboflavin-dependent flavoproteins sustain intracellular redox cycling capacity, and vitamin E halts the chain reaction of lipid peroxidation at the membrane level (23, 26, 27). These processes collectively contribute to mitochondrial stability, redox homeostasis, and the maintenance of neurotrophic signaling—such as SIRT1 and BDNF—pathways that are critical for memory, processing speed, attention, executive function, and semantic fluency (26, 28–30). This implies that cognitive health may be influenced not only by individual nutrients but also by the interactive patterns among them.

However, most population-based studies continue to examine nutrients in isolation, seldom directly testing nutrient-nutrient synergies (31). This approach is suboptimal in practice, as nutrients often coexist within dietary patterns and are closely linked to socioeconomic status and comorbidity profiles (32). Traditional regression models are further limited by high collinearity among nutritional variables (33). Thus, although vitamin B2, copper, and vitamin E have each been linked to mitochondrial support, antioxidative stress, neuroprotection, and neuroinflammatory regulation, it remains unclear which nutrient combinations carry the most informative value for cognitive screening in the community.

In this study, we aim to address this gap. We integrated multidomain cognitive performance (CERAD-WL, DSST, AFT) with sociodemographic, clinical, and dietary nutrient data from the 2011–2014 National Health and Nutrition Examination Survey (NHANES), a nationally representative U.S. health and nutrition surveillance program that systematically captures adults’ health status and dietary intake (34–36). We trained multiple machine learning models and applied SHapley Additive exPlanations (SHAP) to: (i) quantify the relative contribution of established risk factors (age, educational attainment, poverty-income ratio, hypertension, diabetes); (ii) identify nutrition-related predictive features, particularly vitamin B2; and (iii) reveal potential interaction patterns between vitamin B2 and copper and vitamin E. Furthermore, motivated by these model-derived interactions, we conducted exploratory *in vitro* experiments in human SH-SY5Y neuroblastoma cells to examine whether vitamin B2 alone, or in combination with copper or vitamin E, modulates oxidative stress levels and neuroprotective pathways. Taken together, this approach is intended to support a practical early screening strategy for cognitive risk: one that can be used at the community level, is grounded in biology, and also identifies nutritional factors that people could realistically change.

## 2 Materials and methods

### 2.1 Data source and study population

This study used publicly available data from the NHANES, covering two continuous cycles (2011–2014, total sample *N* = 19,931). NHANES employs a multistage, stratified probability sampling design and is nationally representative of the non-institutionalized U.S. population’s health and nutritional status.

**Figure 1:**
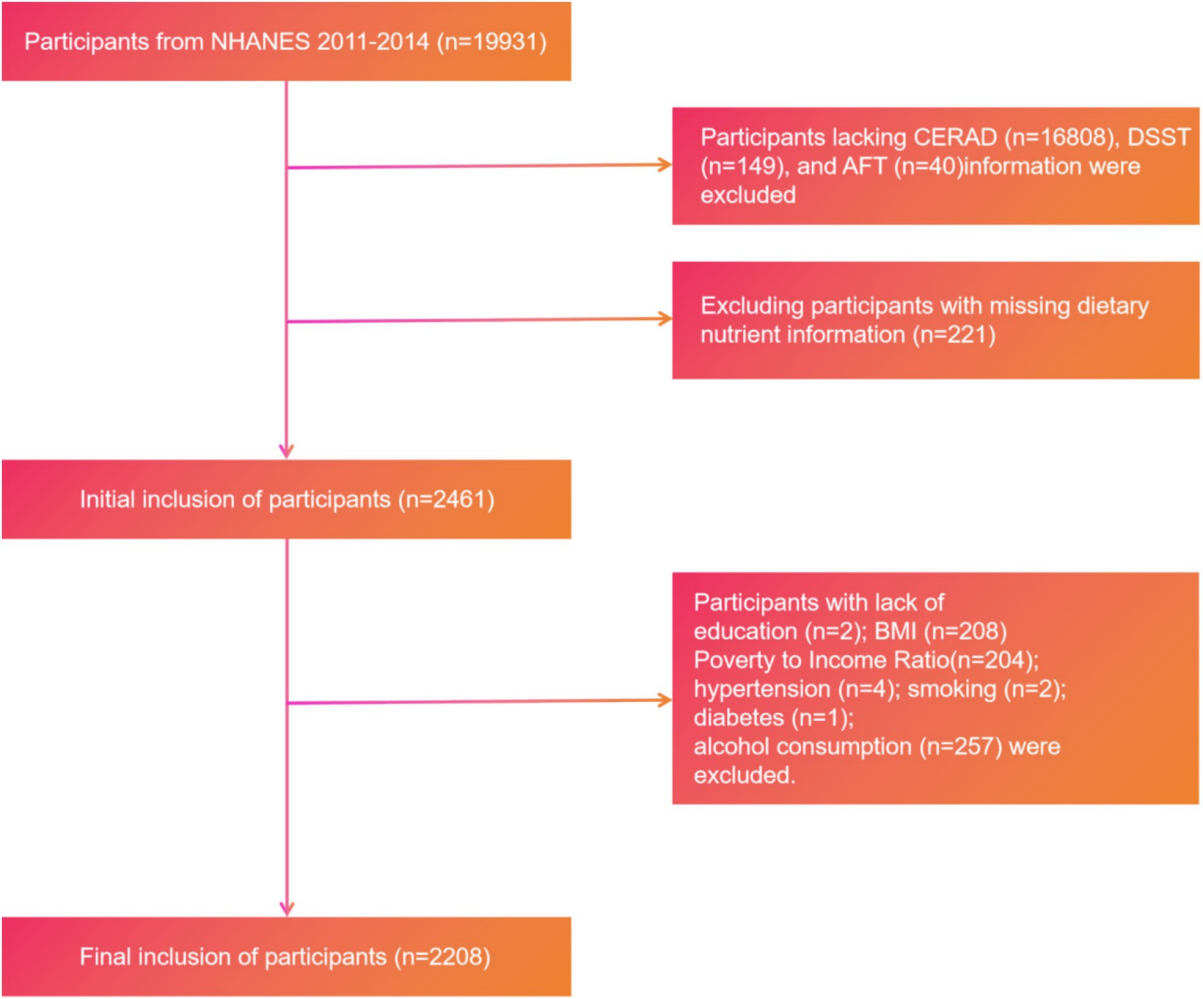
Study participant selection flowchart.

Layered inclusion/exclusion strategy:

1. Cognitive tests: Required complete results for CERAD-WL, DSST, and AFT.
2. Dietary data: Required a 24-h dietary recall. Participants missing any cognitive test or dietary intake were excluded, yielding the initial analytic sample *N* = 2,461.
3. Predictor set for community applicability: To simplify the model and enhance generalizability, predictors were limited to interview-obtainable covariates. Exclusions at this step were triggered by missing data in the covariates shown in Figure 1 (education, PIR, BMI, hypertension, smoking, diabetes, alcohol use), removing *n* = 253 and resulting in the final sample *N* = 2,208.

The sample size at each step and participant flow are shown in Figure 1. Reporting follows STROBE/TRIPOD recommendations (37, 38).

As NHANES data are publicly available and fully de-identified, and its study protocol was approved by the NCHS Research Ethics Review Board, this secondary data analysis did not require additional ethical approval (39).

### 2.2 Outcome definition: cognitive impairment

In NHANES 2011–2014, cognition was assessed with three brief screening tests—CERAD, DSST, and AFT—which sample distinct domains (episodic memory, processing speed/executive attention, and semantic fluency, respectively) (15–17). Consistent with NHANES analytic/reporting practice and prior NHANES studies, we operationalized low cognitive performance per test as a score at or below the 25th percentile of that test’s distribution in our analytic sample (40). We chose this threshold *a priori* based on the existing literature, where the lowest-quartile rule is commonly used to flag low performance in population-based screening contexts and to maintain comparability across sociodemographic strata in a multiethnic survey (41). These instruments are intended for epidemiologic screening rather than clinical diagnosis, and fixed raw-score cutoffs vary by age and education without a single standard applicable to the multiethnic NHANES population; therefore, we did not apply diagnostic cutoffs. Analyses were conducted separately for each test-defined outcome.

### 2.3 Dietary intake assessment

Dietary intake was assessed using two 24-h dietary recall interviews recalls administered with the USDA Automated Multiple-Pass Method. Each recall covered the day before the interview (00:00 – 24:00; midnight-to-midnight). The first recall was conducted in person at the Mobile Examination Center, and the second was completed via telephone interview 3–10 days later. To approximate usual intake, we used the mean of the two recalls; when only one recall was available, the Day 1 value was used instead (42). In our analytic sample, 92.35% of participants completed one recall and 7.65% completed two recalls.

A total of 48 dietary nutrients were included in the analysis, covering energy intake, macronutrients, micronutrients, and other dietary components. Specifically, the categories were as follows: (1) Energy intake: total energy; (2) Macronutrients: protein, carbohydrates, total sugars, dietary fiber, total fat, saturated fatty acids, monounsaturated fatty acids, polyunsaturated fatty acids, and cholesterol; (3) Vitamins: vitamin A and its subtypes (retinol, *α*-carotene, *β*-carotene, β-cryptoxanthin), vitamin D (D2 and D3), vitamin E (as α-tocopherol), vitamin K, vitamin C, and B-complex vitamins (vitamin B1, B2, B6, B12, niacin, folate and its forms, and total choline); (4) Minerals: calcium, phosphorus, magnesium, iron, zinc, copper, sodium, potassium, and selenium; (5) Other dietary components: caffeine, theobromine, alcohol, and moisture intake (including plain water, other beverages, and the water content of foods—assessed by a 24-h dietary recall). All nutrient intake levels were expressed as mean ± standard deviation (Mean ± SD) to describe the distribution characteristics of dietary intake in the population.

### 2.4 Model development and evaluation

We developed predictive models using nationally representative cross-sectional data from the 2011–2014 NHANES cycles (initial sample size *N* = 19,931). We first restricted the sample to participants who had completed all three cognitive assessments (CERAD-WL, DSST, and AFT) and who had at least one valid 24-h dietary recall interview, yielding 2,461 eligible participants. We then further required complete information on key interview-based sociodemographic and clinical variables (including age, sex, race/ethnicity, education, PIR, BMI, smoking status, drinking status, and self-reported diabetes and hypertension), resulting in a final analytic sample of *N* = 2,208.

To enhance feasibility in community screening and routine clinical encounters, we intentionally limited candidate predictors to variables that can be obtained through brief interview or standard self-report (sociodemographic characteristics, lifestyle behaviors, and common chronic conditions), supplemented by nutrient intake derived from 24-h dietary recall. All continuous predictors were standardized using z-score transformation (subtracting the training-fold mean and dividing by the training-fold standard deviation), and the same scaling parameters were applied to the corresponding validation/test folds during cross-validation. This approach was used to improve numerical stability across variables with different scales and to prevent information leakage. To reduce redundancy and multicollinearity among predictors, we computed variance inflation factors (VIFs) and iteratively removed predictors with VIF > 3, refitting after each step, in line with established guidance on handling multicollinearity (43).

Because cognitive impairment represented the minority class, we addressed class imbalance within each training fold using a combination of synthetic minority oversampling (SMOTE) and random undersampling of the majority class (44). This strategy was intended to improve the model’s ability to identify cognitively impaired individuals.

We compared seven machine learning models with diverse inductive biases and algorithmic characteristics: (1) Random Forest, (2) LightGBM, (3) K-Nearest Neighbors (K-NN), (4) Naive Bayes, (5) Support Vector Machine (SVM) and (6) XGBoost. All models followed a unified training procedure: (i) stratified 5-fold cross-validation was applied to preserve the proportion of cognitively impaired vs. non-impaired cases in each fold, thereby improving the stability and representativeness of performance estimates; and (ii) hyperparameters were optimized using grid search.

To comprehensively assess predictive performance, we adopted a multidimensional evaluation framework: (1) Discriminative ability was quantified using overall accuracy and the area under the receiver operating characteristic curve (AUC-ROC); (2) Clinical utility was evaluated using sensitivity and specificity, reflecting the model’s ability to correctly identify true positives (cognitive impairment) and exclude true negatives (no impairment), respectively; and (3) Predictive reliability was evaluated using the F*β* score and the area under the precision–recall curve (AUC-PR), both of which are particularly informative in class-imbalanced settings. The Fβ score balances precision and recall (with β = 1 corresponding to the F1 score), while the AUC-PR reflects the stability of positive-class detection in the minority (impaired) group (45).

### 2.5 Model explainability

In terms of model interpretation, we conducted an in-depth analysis of the best-performing model using SHAP (46). By calculating SHAP values for each sample, we generated three core outputs: (1) a global feature importance ranking based on the mean absolute SHAP values; (2) individualized explanation plots (force plots) illustrating the key drivers of individual predictions; and (3) an interaction effect heatmap revealing relationships between features. All analyses were performed in a Python 3.8 environment. Machine learning models were implemented using scikit-learn version 1.0.2, SHAP analyses were conducted using version 0.41.0, and visualizations were generated with Matplotlib 3.5 and Seaborn 0.11. The overall analytical pipeline—including characterization of missingness using Little’s MCAR test, applying an individual-level complete-case strategy within each analysis (i.e., excluding only participants missing a required variable rather than discarding the variable itself), controlling multicollinearity via VIF and confirming feature contribution using Boruta, performing z-score standardization of continuous variables based on parameters estimated from the training set, and interpreting the final models using SHAP—was predefined prior to model training. This design was intended to obtain stable and clinically meaningful predictions without relying on the assumption that individual predictors follow a normal distribution. The study followed the TRIPOD guidelines to ensure completeness and transparency in reporting the prediction model (38).

### 2.6 *In vitro* validation of key nutrient effects

Human SH-SY5Y neuroblastoma cells cell lines were purchased from the Type Culture Collection of the Chinese Academy of Sciences (Shanghai, China) (47). Cells were cultured in DMEM/F12 (1:1, Gibco, Cat#11220033) + 10% heat-inactivated FBS (FBS, H clone, Cat# SV30087.03) + 1% penicillin at 37 °C, 5% CO₂, and used at passages 5–20. To validate model-motivated neuroprotective hypotheses, cells were pre-incubated for 6 h with vitamin B2 (5 μM), vitamin E (20 μM), and/or CuSO₄ (5 μM) (48–50). Unless otherwise specified, pretreatments were maintained during the oxidative challenge to mimic continuous exposure.

Cells were pre-loaded with DCFH-DA (10 μM, 30 min, 37 °C, serum-free, protected from light), washed twice with PBS, and transferred to phenol-red- and pyruvate-free medium. Oxidative stress was then elicited by freshly diluting H_2_O_2_ to 200 μM and incubating for 2 h (no wash after addition) (51). Fluorescence (Ex/Em 485/535 nm) was recorded on a plate reader either kinetically (every 2–5 min for 30–60 min) or at a fixed endpoint (15–30 min). Background (dye-free wells) was subtracted, and signals were normalized to cell content by SRB staining or to total protein by BCA in matched wells. t-BHP (50–100 μM) served as a positive control in selected runs. Non-cytotoxicity at working doses was verified on parallel plates using CCK-8. Where indicated, NAC (2–5 mM) or catalase (200–500 U/mL) were included as antioxidant controls to confirm ROS-dependent signals.

Total RNA was extracted with TRIzol and treated with RNase-free DNase I. RNA quantity and purity were assessed (A260/280 ≈ 2.0; A260/230 > 1.8). One microgram of RNA was reverse-transcribed with random hexamers; minus-RT controls (no reverse transcriptase) were prepared in parallel. RT-qPCR was performed using SYBR Green chemistry in 10–20 μL reactions (primer 0.2–0.5 μM; 10–50 ng cDNA per well) on a 96-well instrument with the following program: 95 °C 2 min; 40 cycles of 95 °C 15 s and 60 °C 30 s; followed by a melt curve (65–95 °C, 0.5 °C increments). No-template controls (NTC) were included for each primer pair. Primer specificity and efficiency were verified by single-peak melt curves, a single band of the expected size (agarose gel), and 5-point standard curves (efficiency 90–110%, R^2^ ≥ 0.99). GAPDH served as the reference gene (stability verified under the present treatments); relative expression was calculated by 2^-ΔΔCt. Human primer sequences were: SIRT1 F: TAGACACGCTGGAACAGGTTGC, R: CTCCTCGTACAGCTTCACAGTC; BDNF (exon IX, total) F: ACTCTGGAGAGCGTGAATGG, R: CGTAGAAGTATTGCTTCAGTTGG; GAPDH F: GTCTCCTCTGACTTCAACAGCG, R: ACCACCCTGTTGCTGTAGCCAA (52, 53). Experiments used ≥3 biologically independent replicates, each measured in technical triplicate (technical replicates averaged per sample).

### 2.7 Statistical analysis

We performed all statistical analyses to compare baseline characteristics between participants with and without cognitive impairment. Continuous variables were summarized as mean ± standard deviation (Mean ± SD), and categorical variables were summarized as counts and percentages [*n* (%)]. Between-group comparisons were conducted using: (i) chi-square tests for categorical variables; (ii) t-tests or analysis of variance (ANOVA) for continuous variables that were approximately normally distributed; and (iii) Kruskal–Wallis *H* tests for continuous variables that were not normally distributed.

To compare predictive performance across machine learning models, we analyzed model evaluation metrics (accuracy, Fβ score, sensitivity, specificity, AUC-PR, and AUC-ROC) across models. For metrics that were approximately normally distributed, we applied one-way ANOVA; for metrics that were non-normally distributed, we used the Kruskal–Wallis H test. When an overall difference across models was detected (*p* < 0.05), we performed *post hoc* pairwise comparisons with appropriate correction for multiple testing (Tukey’s honestly significant difference test following ANOVA, or Dunn-type procedures following Kruskal–Wallis).

For *in vitro* experiments, data from at least three biologically independent replicates are presented as mean ± standard deviation (Mean ± SD). Group differences were assessed using one-way ANOVA followed by Tukey’s post hoc test.

## 3 Results

### 3.1 Baseline characteristics of participants

A total of 2,208 participants were included in this study, among whom 1,084 (49.09%) were female, with a mean age of 69.23 ± 6.71 years. The baseline characteristics of the participants, grouped by cognitive status based on the AFT, are detailed in Table 1. DSST and CERAD-WL by group are provided in Supplementary Tables 1, 2.

**Table 1:** Baseline characteristics of participants by AFT.

Characteristic	Overall *N* = 2,208	No cognitive impairment *N* = 1,951	Cognitive impairment *N* = 257	*p*
Age(year)^a^, Mean ± SD	69.23 ± 6.71	69.01 ± 6.67	70.90 ± 6.78	<0.001
Sex^b^, *n* (%)				0.151
Female	1,084 (49.09%)	947 (48.54%)	137 (53.31%)	
Male	1,124 (50.91%)	1,004 (51.46%)	120 (46.69%)	
Race/ethnicity^b^, *n* (%)				<0.001
Mexican	187 (8.47%)	166 (8.51%)	21 (8.17%)	
Other Hispanic	217 (9.83%)	180 (9.23%)	37 (14.40%)	
Non-Hispanic White	1,127 (51.04%)	1,054 (54.02%)	73 (28.40%)	
Non-Hispanic Black	490 (22.19%)	393 (20.14%)	97 (37.74%)	
Other race	187 (8.47%)	158 (8.10%)	29 (11.28%)	
Education^b^, *n* (%)				<0.001
Less than 9th	221 (10.01%)	173 (8.87%)	48 (18.68%)	
9th–11th	278 (12.59%)	225 (11.53%)	53 (20.62%)	
High school	516 (23.37%)	439 (22.50%)	77 (29.96%)	
Some college	653 (29.57%)	604 (30.96%)	49 (19.07%)	
College graduate	540 (24.46%)	510 (26.14%)	30 (11.67%)	
Poverty-to-Income Ratio^a^, Mean ± SD	2.67 ± 1.61	2.75 ± 1.61	2.05 ± 1.46	<0.001
BMI^a^, Mean ± SD	29.19 ± 6.40	29.23 ± 6.45	28.86 ± 6.05	0.573
Smoking status^b^, *n* (%)				0.481
No	1,929 (87.36%)	1,708 (87.54%)	221 (85.99%)	
Yes	279 (12.64%)	243 (12.46%)	36 (14.01%)	
Drinking status^b^, *n* (%)				<0.001
No	354 (16.03%)	289 (14.81%)	65 (25.29%)	
Yes	1,854 (83.97%)	1,662 (85.19%)	192 (74.71%)	
Diabetes ^b^, *n* (%)				0.005
No	1,693 (76.68%)	1,514 (77.60%)	179 (69.65%)	
Yes	515 (23.32%)	437 (22.40%)	78 (30.35%)	
Hypertension ^b^, *n* (%)				0.007
No	839 (38.00%)	761 (39.01%)	78 (30.35%)	
Yes	1,369 (62.00%)	1,190 (60.99%)	179 (69.65%)	
Energy^a^, Mean ± SD	1,870.76 ± 790.66	1,897.82 ± 783.62	1,665.30 ± 814.86	<0.001
Protein^a^, Mean ± SD	73.26 ± 35.91	74.08 ± 35.64	67.08 ± 37.44	<0.001
Carbohydrate^a^, Mean ± SD	226.06 ± 99.54	228.73 ± 99.76	205.75 ± 95.65	<0.001
Total Sugar^a^, Mean ± SD	96.36 ± 59.86	97.98 ± 60.84	84.10 ± 50.20	<0.001
Dietary fiber^a^, Mean ± SD	17.15 ± 10.35	17.38 ± 10.39	15.42 ± 9.87	0.001
Total Fat^a^, Mean ± SD	72.07 ± 39.51	73.41 ± 39.31	61.89 ± 39.57	<0.001
Saturated fatty acids^a^, Mean ± SD	22.72 ± 13.72	23.22 ± 13.71	18.99 ± 13.20	<0.001
Monounsaturated fatty acids^a^, Mean ± SD	25.77 ± 15.17	26.16 ± 15.03	22.74 ± 15.84	<0.001
Polyunsaturated fatty acids^a^, Mean ± SD	17.40 ± 11.32	17.75 ± 11.42	14.74 ± 10.21	<0.001
Cholesterol^a^, Mean ± SD	268.23 ± 215.20	271.42 ± 215.12	244.01 ± 214.69	0.003
Vitamin E as alpha-tocopherol^a^, Mean ± SD	8.32 ± 6.62	8.48 ± 6.75	7.06 ± 5.30	<0.001
Alpha-tocopherol^a^, Mean ± SD	0.79 ± 3.92	0.81 ± 4.05	0.64 ± 2.83	0.535
Retinol^a^, Mean ± SD	421.62 ± 718.21	430.57 ± 751.47	353.72 ± 374.55	<0.001
Vitamin A^a^, Mean ± SD	657.99 ± 936.89	670.88 ± 973.68	560.08 ± 578.70	<0.001
Alpha-carotene^a^, Mean ± SD	444.98 ± 1,807.94	457.91 ± 1,889.89	346.84 ± 980.94	0.008
Beta-carotene^a^, Mean ± SD	2,568.05 ± 6,372.00	2,610.54 ± 6,566.52	2,245.51 ± 4,633.78	<0.001
Beta-cryptoxanthin^a^, Mean ± SD	105.26 ± 610.13	102.95 ± 626.78	122.73 ± 465.30	0.121
Lycopene^a^, Mean ± SD	4,479.47 ± 8,793.72	4,527.35 ± 8,699.66	4,115.98 ± 9,487.66	0.002
Lutein+zeaxanthin^a^, Mean ± SD	1,764.40 ± 4,054.16	1,793.08 ± 4,153.37	1,546.72 ± 3,200.68	0.005
Thiamin(Vitamin B1) ^a^, Mean ± SD	1.48 ± 0.74	1.50 ± 0.74	1.34 ± 0.73	<0.001
Riboflavin(Vitamin B2)^a^, Mean ± SD	1.96 ± 1.10	2.00 ± 1.11	1.67 ± 0.92	<0.001
Niacin^a^, Mean ± SD	22.65 ± 12.61	22.94 ± 12.72	20.47 ± 11.46	<0.001
Vitamin B6^a^, Mean ± SD	1.94 ± 1.33	1.96 ± 1.35	1.78 ± 1.16	0.015
Total folate^a^, Mean ± SD	381.07 ± 233.87	388.43 ± 239.62	325.21 ± 175.04	<0.001
Folic acid^a^, Mean ± SD	166.34 ± 165.72	170.13 ± 170.65	137.55 ± 118.37	0.008
Food folate^a^, Mean ± SD	214.77 ± 145.70	218.33 ± 149.33	187.71 ± 111.04	<0.001
Folate(DFE)^a^, Mean ± SD	497.50 ± 332.90	507.49 ± 341.57	421.66 ± 245.35	<0.001
Total choline^a^, Mean ± SD	314.84 ± 176.11	317.69 ± 175.65	293.27 ± 178.37	0.002
Vitamin B12^a^, Mean ± SD	4.74 ± 7.96	4.85 ± 8.36	3.86 ± 3.52	<0.001
Added vitamin B12^a^, Mean ± SD	0.95 ± 2.35	0.98 ± 2.44	0.70 ± 1.51	0.086
Vitamin C^a^, Mean ± SD	83.23 ± 89.63	82.84 ± 88.48	86.16 ± 98.07	0.748
Vitamin K^a^, Mean ± SD	128.23 ± 373.60	131.62 ± 392.81	102.51 ± 165.00	<0.001
Calcium^a^, Mean ± SD	846.94 ± 496.98	858.69 ± 491.71	757.71 ± 527.81	<0.001
Phosphorus^a^, Mean ± SD	1,237.55 ± 566.88	1,254.09 ± 560.99	1,112.01 ± 596.07	<0.001
Magnesium^a^, Mean ± SD	286.41 ± 137.56	290.13 ± 138.04	258.12 ± 130.70	<0.001
Iron^a^, Mean ± SD	14.03 ± 8.12	14.23 ± 8.20	12.54 ± 7.30	<0.001
Zinc^a^, Mean ± SD	10.05 ± 5.92	10.24 ± 6.02	8.67 ± 4.91	<0.001
Copper^a^, Mean ± SD	1.24 ± 1.33	1.26 ± 1.39	1.09 ± 0.67	<0.001
Sodium^a^, Mean ± SD	3,105.00 ± 1,436.38	3,142.06 ± 1,420.52	2,823.63 ± 1,525.28	<0.001
Potassium^a^, Mean ± SD	2,575.39 ± 1,188.65	2,609.84 ± 1,188.69	2,313.90 ± 1,157.55	<0.001
Selenium^a^, Mean ± SD	103.77 ± 60.86	105.12 ± 60.98	93.51 ± 59.02	<0.001
Caffeine^a^, Mean ± SD	153.50 ± 177.47	159.87 ± 181.29	105.15 ± 136.08	<0.001
Theobromine^a^, Mean ± SD	32.41 ± 79.24	33.11 ± 81.07	27.10 ± 63.53	0.008
Alcohol^a^, Mean ± SD	7.57 ± 21.22	7.82 ± 21.04	5.65 ± 22.51	0.002
Moisture^a^, Mean ± SD	2,622.60 ± 1,232.44	2,667.62 ± 1,244.03	2,280.86 ± 1,083.27	<0.001
Vitamin D (D2 + D3) ^a^, Mean ± SD	4.60 ± 5.14	4.63 ± 5.14	4.36 ± 5.15	0.096

^a^Student *t*-test, ^b^Chi-square test, SD: standard deviation.

### 3.2 AFT

#### 3.2.1 Model discriminative performance

**Figure 2:**
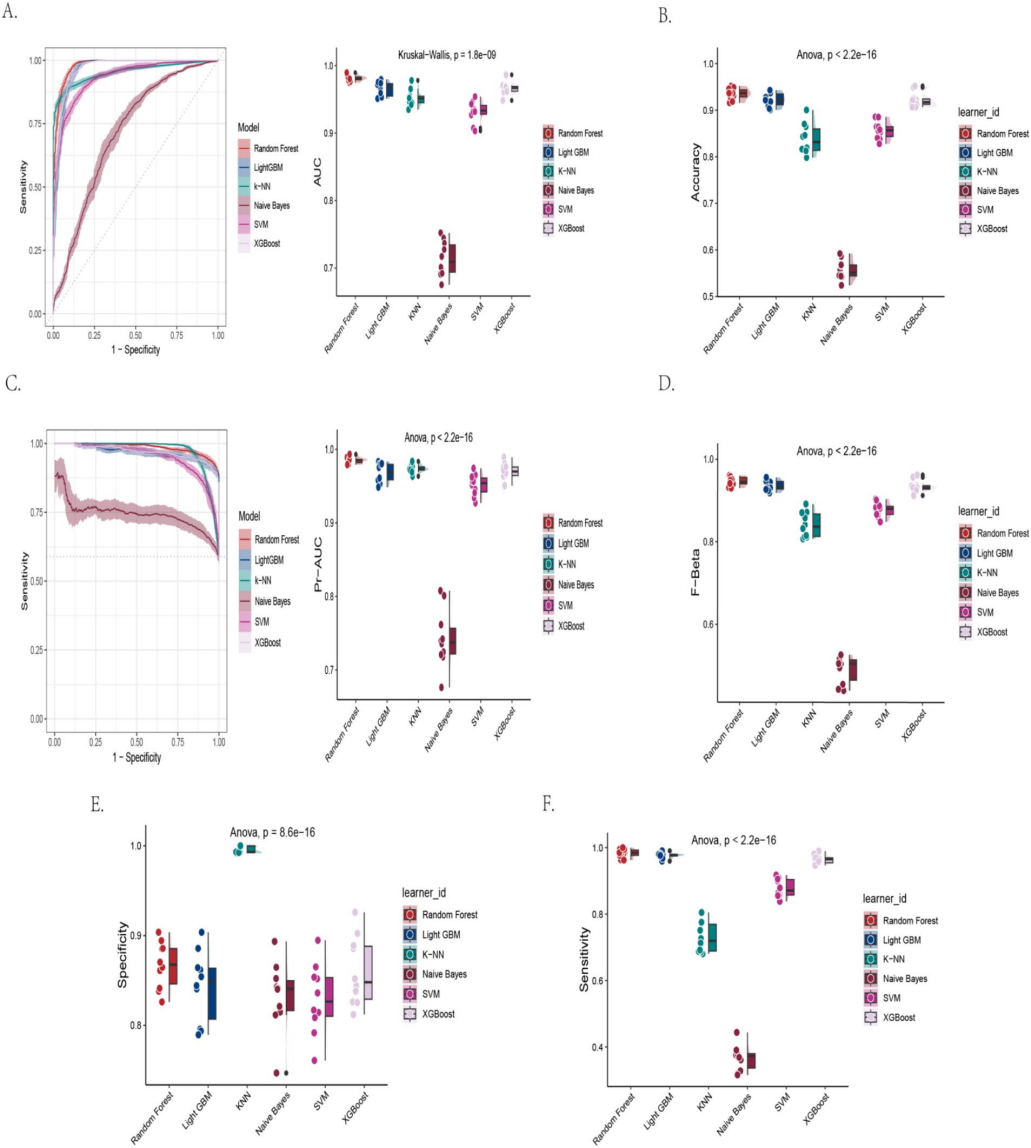
Machine learning model performance on AFT-based cognitive impairment prediction. **(A)** Receiver operating characteristic curve with area under the curve values. **(B)** Classification accuracy across models. **(C)** Area under the precision-recall curve. **(D)** Fβ score (*β* = 1) comparison. **(E)** Model specificity in identifying healthy controls. **(F)** Model sensitivity in detecting cognitive impairment.

This study systematically evaluated the predictive performance of seven machine learning models for cognitive impairment using the AFT (Table 2; Figures 2A–F). The results showed that the Random Forest model demonstrated optimal overall performance, achieving an accuracy of 93.5%, an AUC-ROC of 0.981, while maintaining high sensitivity (0.983) and specificity (0.867). Both LightGBM and XGBoost models also exhibited excellent performance, with accuracy reaching 92.2% and AUC exceeding 0.965, though with slightly lower specificity. While the K-NN model achieved the best specificity (0.996), its overall predictive efficacy was compromised by relatively low sensitivity (0.729). The Naive Bayes model performed poorly, with accuracy of only 55.7% and sensitivity as low as 0.366. SVM demonstrated moderate but stable predictive performance (85.7% accuracy). All inter-model performance differences were statistically significant (*p* < 0.001). Comprehensive analysis indicates that ensemble learning methods (particularly Random Forest) achieved the best balance between sensitivity and specificity, providing a reliable algorithmic choice for AFT-based cognitive impairment screening.

**Table 2:** Performance comparison of seven machine learning models in predicting cognitive impairment based on the AFT.

Model	Accuracy	*F* beta	Area under the ROC curve	Sensitivity	Specificity	Area under the PR curve
Random Forest	0.935	0.946	0.981	0.983	0.867	0.985
Light GBM	0.922	0.936	0.965	0.976	0.843	0.968
K-KNN	0.838	0.841	0.952	0.729	0.996	0.974
Naive Bayes	0.557	0.492	0.714	0.366	0.833	0.743
SVM	0.857	0.878	0.931	0.878	0.829	0.951
XGBoost	0.922	0.935	0.966	0.965	0.860	0.969
*p*	<0.001^a^	<0.001^a^	<0.001^b^	<0.001^a^	<0.001^a^	<0.001^a^

^a^ANOVA test; ^b^Kruskal–Wallis.

#### 3.2.2 Variable importance and interpretation

##### 3.2.2.1 Bee swarm plot

**Figure 3:**
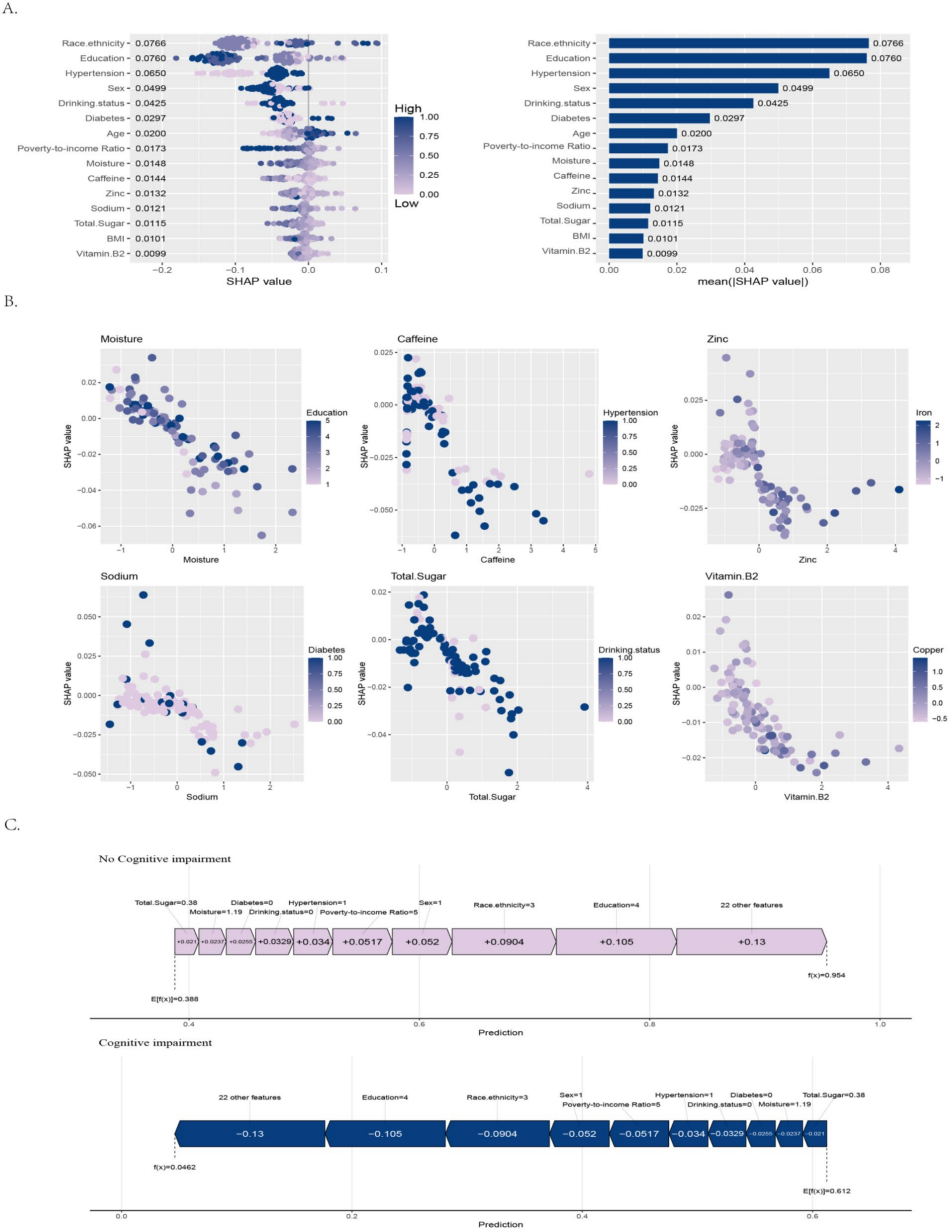
SHAP analysis for cognitive impairment prediction using the AFT. **(A)** Top 15 features ranked by mean absolute SHAP values. **(B)** Dependence plots illustrating nonlinear relationships and interactions between key nutritional predictors and SHAP values. **(C)** Individual prediction explanation using force plots.

In the XGBoost classification model for the AFT, SHAP analysis identified the top 15 features most strongly associated with predicted cognitive impairment outcomes (Figure 3A), ranked by descending mean SHAP values: Race/Ethnicity 0.0766; Education 0.0760; Hypertension 0.0650; Sex 0.0499; Drinking status 0.0425; Diabetes 0.0297; Age 0.0200; PIR 0.0173; Moisture 0.0148; Caffeine 0.0144; Zinc 0.0132; Sodium 0.0121; Total sugar 0.0115; BMI 0.0101; Vitamin B2 0.0099.

The results indicated that race/ethnicity and education level had the strongest associations with model predictions, with contributions greater than those of other factors. Hypertension, sex, and drinking status formed the second group of variables with notable associations. Among metabolic-related factors, diabetes showed stronger associations than BMI, while nutritional factors overall contributed smaller associations, with moisture and caffeine being most prominent. For sodium and total sugar, higher values predominantly show negative SHAP contributions, indicating lower predicted impairment risk after adjustment for covariates; this pattern is consistent with Table 1, where participants with cognitive decline exhibit lower mean sodium and total sugar. Overall, AFT scores were not only associated with nutritional intake patterns but also with socioeconomic status and chronic disease conditions, emphasizing the influence of multiple factors.

##### 3.2.2.2 Dependency plot

The SHAP analysis based on the XGBoost model (Figure 3B) indicated differential associations of nutrition-related variables with predicted cognitive impairment. Moisture, caffeine, zinc, sodium, total sugar, and vitamin B2 showed negative correlation trends with predicted risk. The association between higher moisture intake and lower predicted risk appeared stronger in participants with lower education levels. Zinc demonstrated an interaction with iron: lower zinc was associated with higher predicted risk when iron was also low, whereas higher zinc was associated with lower predicted risk when iron was high. In hypertensive participants, the association between caffeine intake and lower predicted risk was more evident. Among diabetic participants, lower sodium intake was associated with higher predicted risk, while the association was weaker in non-diabetic individuals. In alcohol-consuming participants, higher sugar intake was paradoxically associated with lower predicted risk. The association between vitamin B2 and lower predicted risk appeared stronger under higher copper levels.

##### 3.2.2.3 Force plot

Analysis based on SHAP force plots showed that under AFT assessment (Figure 3C), the model predicted a 95.4% probability of no cognitive impairment for this individual, which was substantially higher than the baseline probability of 38.8%. Among the important features associated with this prediction, education level (+0.105), race (+0.0904), and gender (+0.052) were the main contributors toward predictions of normal cognition. The predicted probability of cognitive impairment was only 4.62%, below the baseline probability of 61.2%. Notably, the contribution of this individual’s nutritional characteristics (including vitamin B12 and zinc levels) accounted for less than 5% of the model output, suggesting that in individuals with favorable sociodemographic profiles, the influence of nutritional factors may be comparatively smaller relative to higher-level social determinants.

### 3.3 CERAD-WL

#### 3.3.1 Model discrimination performance

**Figure 4:**
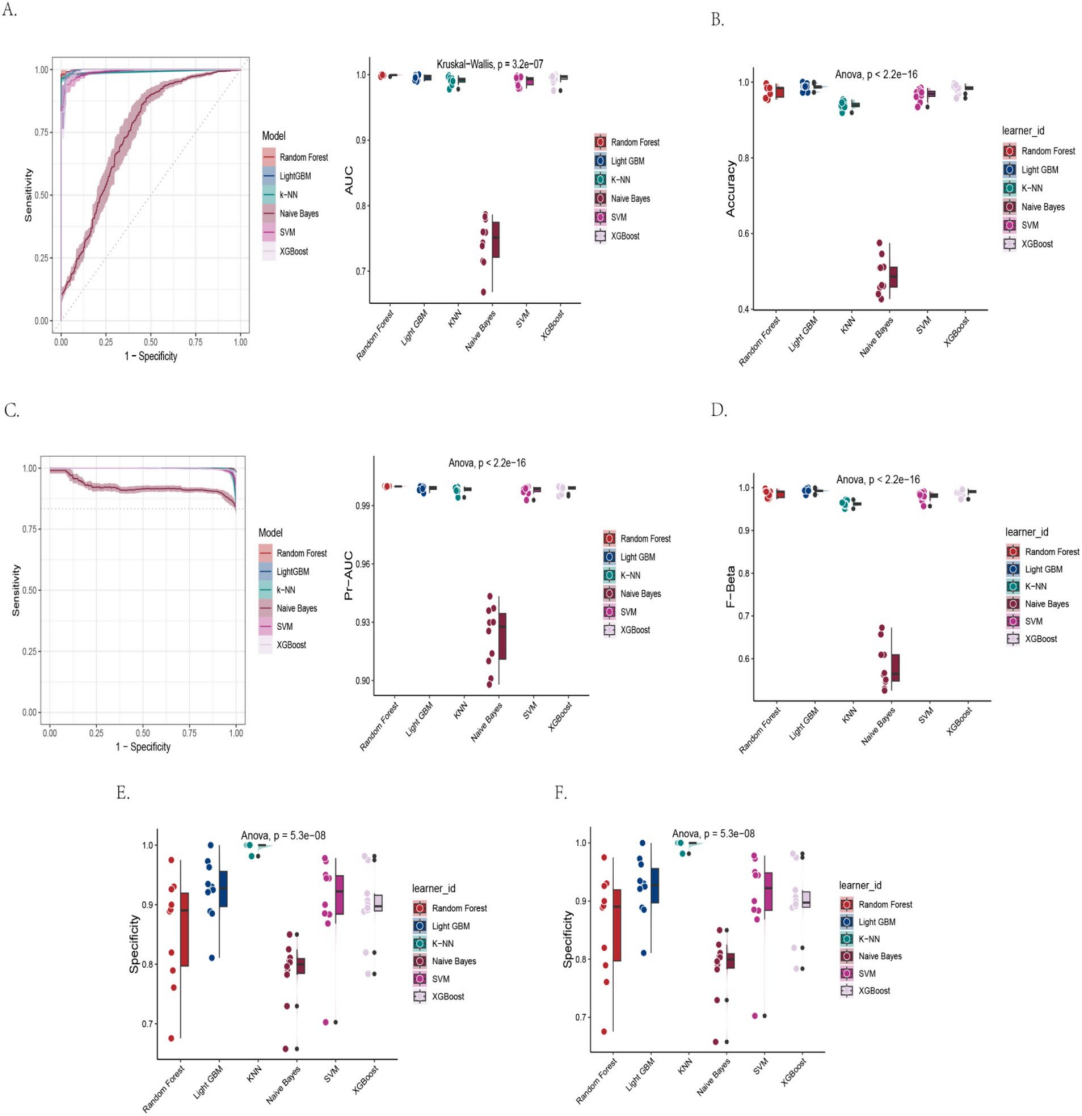
Performance metrics of machine learning models for CERAD-WL-based cognitive impairment detection. **(A)** Receiver operating characteristic curve with area under the curve values. **(B)** Classification accuracy across models. **(C)** Area under the precision-recall curve. **(D)** F_β_ score (*β* = 1) comparison. **(E)** Model specificity in identifying healthy controls. **(F)** Model sensitivity in detecting cognitive impairment.

Baseline characteristics of participants grouped by CERAD-WL cognitive status are provided in Supplementary Table 1. Multiple machine learning models were constructed based on the CERAD-WL scale to predict the risk of cognitive impairment. The performance metrics of different models are as follows (Table 3; Figures 4A–F).

**Table 3:** Model performance comparison on CERAD-WL cognitive impairment screening.

Model	Accuracy	*F* beta	Area under the ROC curve	Sensitivity	Specificity	Area under the PR curve
Random Forest	0.976	0.986	0.999	1.000	0.856	1.000
Light GBM	0.987	0.992	0.996	0.999	0.923	0.999
K-KNN	0.939	0.962	0.991	0.927	0.998	0.998
Naive Bayes	0.490	0.583	0.745	0.431	0.785	0.922
SVM	0.966	0.979	0.990	0.978	0.903	0.998
XGBoost	0.982	0.989	0.994	0.999	0.896	0.999
*p*	<0.001^a^	<0.001^a^	<0.001^b^	<0.001^a^	<0.001^a^	<0.001^a^

^a^ANOVA test; ^b^Kruskal–Wallis.

Among all models, LightGBM showed the best internal discrimination (AUC-ROC 0.996) and recall of impaired individuals (sensitivity 0.999), while maintaining acceptable specificity (0.923). This indicates that, within NHANES, the model can almost always flag individuals classified as impaired by CERAD-WL while still excluding most unimpaired individuals. The XGBoost model also demonstrated high performance, with all metrics approaching or exceeding 0.98. The Random Forest model achieved an accuracy of 0.976, an AUC-ROC of 0.999, and a sensitivity of 1.000, but its specificity was relatively lower (0.856). The SVM and K-KNN models also performed well, with accuracies of 0.966 and 0.939 and AUC-ROCs of 0.990 and 0.991, respectively. In contrast, the Naive Bayes model performed significantly worse, with an accuracy of only 0.490, a sensitivity of 0.431, and a specificity of 0.785.

The differences in major metrics between models were all statistically significant (*p* < 0.001). Accuracy, F-beta score, sensitivity, specificity, and AUC-PR were tested using ANOVA, while AUC-ROC was tested using the Kruskal–Wallis test. Overall, ensemble algorithms such as LightGBM and XGBoost demonstrated extremely high accuracy and stability in predicting cognitive impairment based on the CERAD-WL test.

#### 3.3.2 Variable importance and interpretation

##### 3.3.2.1 Bee swarm plot

**Figure 5:**
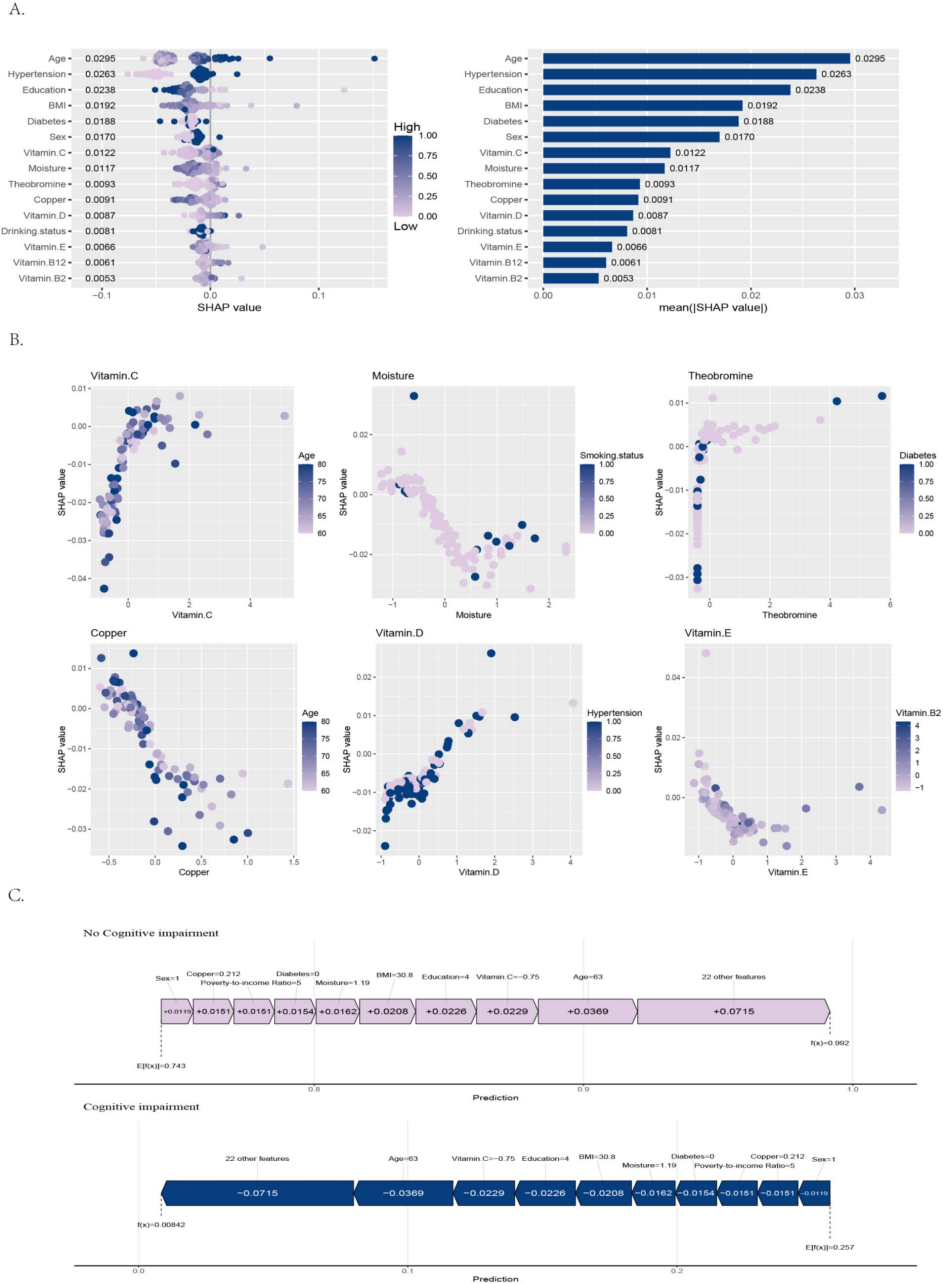
Interpretable machine learning analysis for CERAD-WL-based cognitive impairment prediction. **(A)** Feature importance ranking, **(B)** nonlinear predictor effects and interactions, **(C)** case-specific prediction interpretation.

The SHAP bee swarm plot revealed the relative importance of each variable in the predictive model (Figure 5A). Among all variables, age had the highest mean absolute SHAP value (0.0295), showing the strongest association with model predictions. Hypertension (0.0263) and education (0.0238) also showed notable associations. BMI (0.0192), diabetes (0.0188), and sex (0.0170) demonstrated moderate associations.

Nutrition-related factors, such as vitamin C (0.0122), moisture (0.0117), theobromine (0.0093), copper (0.0091), vitamin D (0.0087), drinking status (0.0081), vitamin E (0.0066), vitamin B12 (0.0061), and vitamin B2 (0.0053), had relatively lower mean SHAP values, indicating smaller but still observable associations with model predictions. Overall, sociodemographic variables-particularly age and hypertension-were the strongest predictors in the model, while various micronutrients also showed contributory associations with the model’s discriminative ability.

##### 3.3.2.2 Dependency plot

Analysis of SHAP dependence plots uncovered complex nonlinear associations between key predictors and predicted cognitive impairment risk (Figure 5B). The relationship between vitamin E and vitamin B2 showed an interaction, where higher vitamin B2 levels were associated with stronger contributions of vitamin E in the model, suggesting a potential synergistic pattern. In hypertensive populations, higher vitamin D levels were more strongly associated with predictions of no cognitive impairment. Other features, such as vitamin C, moisture, theobromine, and copper, showed weaker associations, but their effects varied depending on age, smoking status, or diabetes. Smoking appeared to modify the association of water intake with model predictions, while the SHAP contribution of theobromine was influenced by diabetes status. In older adults, higher copper levels were associated with higher predicted risk, whereas the association of vitamin C with lower predicted risk appeared weaker.

##### 3.3.2.3 Force plot

Using the predictive model based on the CERAD-WL test, we employed SHAP force plots to interpret the predictions for “no cognitive impairment” and “cognitive impairment” in the same subject(Figure 5C).

For the “no cognitive impairment” prediction, the subject’s baseline predicted value (E[f(x)]) was 0.743, and the final predicted value was 0.992, suggesting performance consistent with no cognitive impairment on the CERAD-WL test. The main features associated with this prediction and their corresponding SHAP values were: age, education, BMI, vitamin C, and moisture.

For the “cognitive impairment” prediction, the subject’s baseline predicted value was 0.257, and the final predicted value decreased to 0.00842, indicating that the model predicted a very low probability of cognitive impairment. In this subject, the model yielded highly consistent results across the CERAD-WL test predictions, suggesting normal cognitive function.

### 3.4 DSST

#### 3.4.1 Model discrimination performance

**Figure 6:**
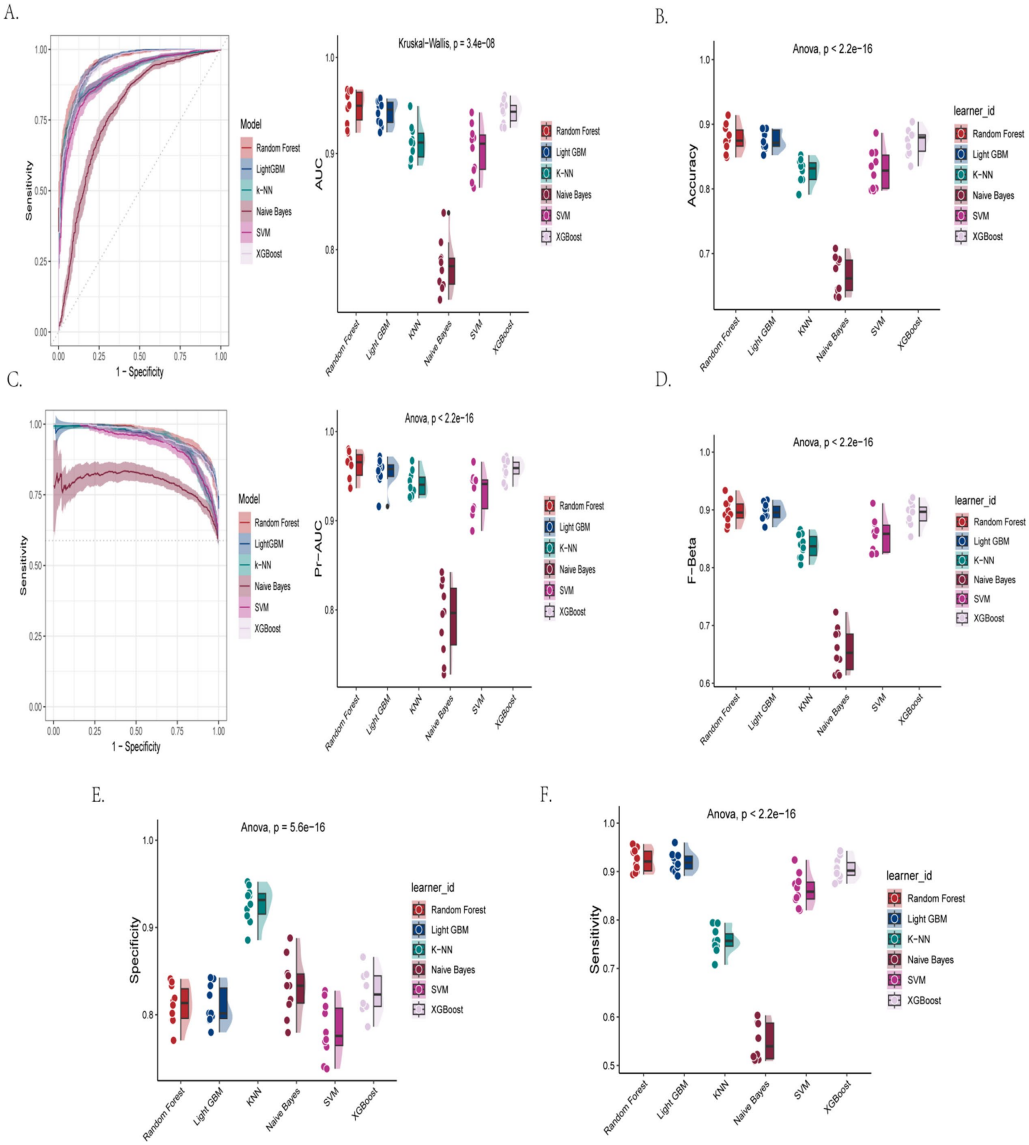
Clinical performance assessment of machine learning models using DSST. **(A)** Diagnostic discrimination (ROC curves), **(B)** overall accuracy, **(C)** positive predictive value (PR curves), **(D)** balanced metric (F1-score), **(E)** healthy control identification, **(F)** cognitive impairment detection.

Participants’ baseline characteristics grouped by DSST cognitive status are provided in Supplementary Table 2. In the prediction of cognitive impairment based on the DSST, various machine learning models demonstrated distinct performance characteristics (Table 4; Figures 6A–F). Ensemble learning methods exhibited superior performance. The Random Forest, LightGBM, and XGBoost models achieved accuracies of 0.877, 0.875, and 0.873, respectively, with AUC-ROC approaching or exceeding 0.94. These models maintained high sensitivity (>0.90; 0.923, 0.920, and 0.906, respectively) while preserving specificity between 0.811–0.826. Furthermore, all three models demonstrated excellent predictive stability, with AUC-PR above 0.95.

**Table 4:** DSST-based cognitive impairment prediction: model performance comparison.

Model	Accuracy	*F* beta	Area under the ROC curve	Sensitivity	Specificity	Area under the PR curve
Random Forest	0.877	0.898	0.948	0.923	0.812	0.962
Light GBM	0.875	0.896	0.942	0.920	0.811	0.954
K-KNN	0.827	0.837	0.911	0.757	0.927	0.941
Naive Bayes	0.666	0.658	0.783	0.550	0.832	0.790
SVM	0.829	0.855	0.903	0.862	0.782	0.931
XGBoost	0.873	0.893	0.943	0.906	0.826	0.957
*p*	<0.001^a^	<0.001^a^	<0.001^b^	<0.001^a^	<0.001^a^	<0.001^a^

^a^ANOVA test; ^b^Kruskal–Wallis.

In contrast, the SVM model showed moderate performance (accuracy = 0.829, AUC-ROC = 0.903, sensitivity = 0.862), albeit with slightly lower specificity (0.782). The K-KNN algorithm achieved comparable accuracy (0.827) but displayed lower sensitivity (0.757) offset by higher specificity (0.927). The Naive Bayes classifier performed substantially worse than other models across all metrics, with particularly low accuracy (0.666), AUC-ROC (0.783), and sensitivity (0.550).

Statistical comparisons revealed significant between-model differences in all performance metrics (*p* < 0.001). Post-hoc analysis for the DSST models revealed that the ensemble methods (Random Forest, LightGBM, XGBoost) did not differ significantly from one another in accuracy or AUC-ROC (*p* > 0.05). However, all three ensemble models significantly outperformed the K-KNN, Naive Bayes, and SVM models (*p* < 0.01 for all pairwise comparisons). The K-KNN and SVM models also performed significantly better than the Naive Bayes model across all primary metrics (*p* < 0.001). These findings collectively demonstrate that ensemble algorithms (Random Forest, LightGBM, XGBoost) provide more accurate and robust prediction of cognitive impairment from DSST performance compared to conventional machine learning approaches.

#### 3.4.2 Variable importance and interpretation

##### 3.4.2.1 Bee swarm plot

**Figure 7:**
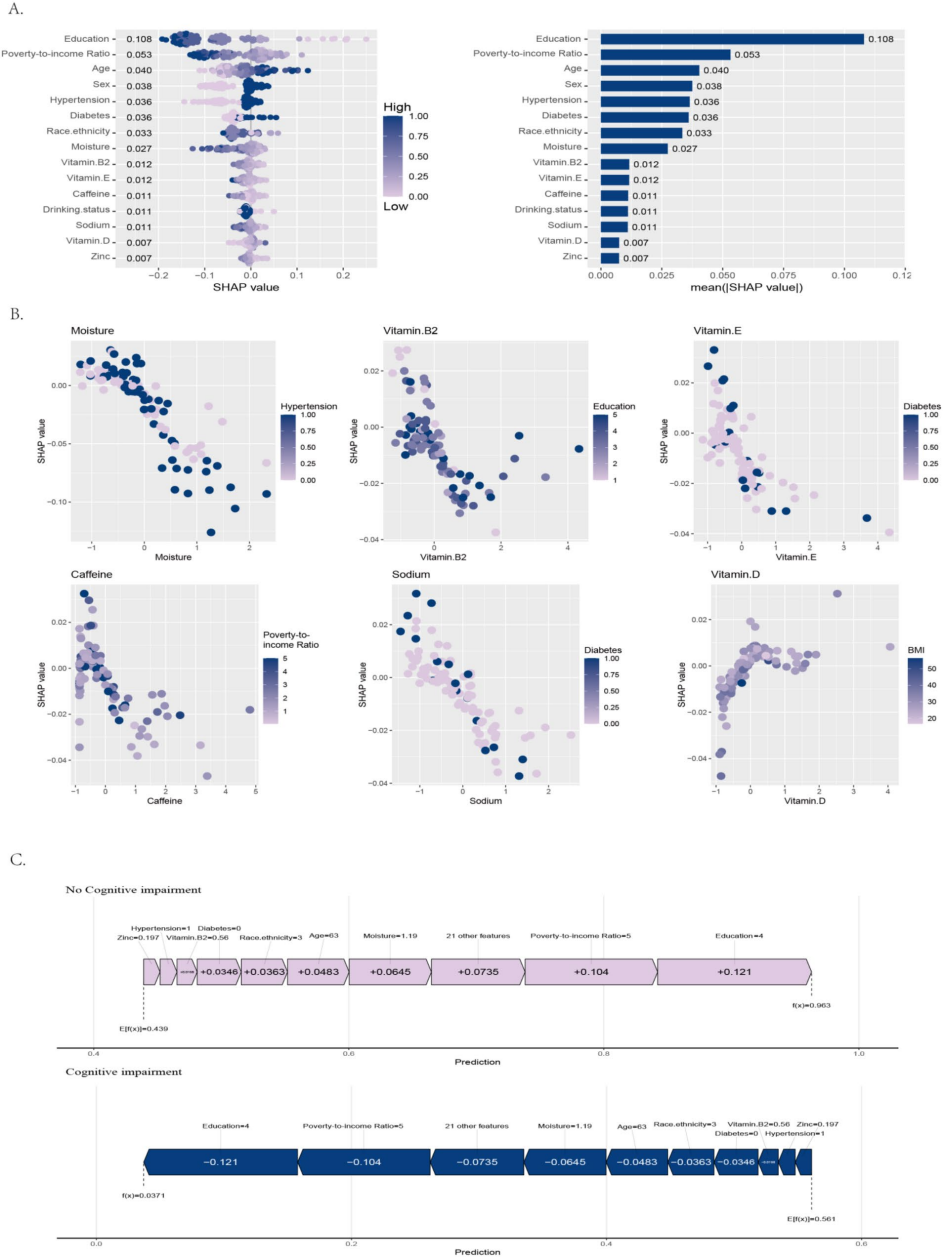
Clinically interpretable features of DSST-based cognitive screening models. **(A)** Key predictor ranking, **(B)** metabolic and sociodemographic interactions, **(C)** individual case risk profiling.

SHAP analysis revealed significant differences in the contributions of various variables to the cognitive impairment prediction model (Figure 7A). Among all features, education had the highest mean absolute SHAP value (0.108), showing the strongest association with model predictions. The PIR (0.053) and age (0.040) also showed strong associations, underscoring the role of socioeconomic status and age in the model’s outputs. In addition, sex (0.038), hypertension (0.036), diabetes (0.036), and race/ethnicity (0.033) were also associated with notable contributions. Moisture (0.027) showed a moderate association.

Nutritional and lifestyle-related variables such as vitamin B2 (0.012), vitamin E (0.012), caffeine (0.011), drinking status (0.011), sodium (0.011), vitamin D (0.007), and zinc (0.007) had relatively lower mean SHAP values, indicating smaller but still observable associations with model predictions. However, these variables may still play a role in shaping predictions for specific individuals.

Overall, the results suggest that socioeconomic and clinical factors were more strongly associated with the model’s predictive outputs, whereas nutritional and lifestyle factors contributed supplementary associations under certain conditions.

##### 3.4.2.2 Dependence plots

The dependence visualization revealed several critical insights into cognitive impairment risk prediction (Figure 7B). SHAP dependence analysis showed that higher levels of moisture were associated with lower predicted cognitive impairment risk. Moreover, in individuals with hypertension, an interaction was observed between moisture and hypertension, with stronger associations in model predictions. Vitamin E also showed an interaction with diabetes, contributing jointly to prediction patterns among diabetic individuals. Vitamin B2 showed an overall negative association with predicted cognitive impairment risk, with stronger associations in individuals with lower education levels, while higher education appeared to attenuate the independent contribution of vitamin B2. Higher caffeine intake was associated with lower predicted risk, and this association was more evident in lower-income groups, suggesting an interaction between caffeine intake and family income. Additionally, higher sodium levels were associated with lower predicted risk, with this association being more prominent in diabetic participants. The association of vitamin D with lower predicted risk was strongest in individuals with moderate BMI, while higher BMI appeared to weaken this association, suggesting possible interactions between nutritional factors and metabolic status.

Overall, these results indicate that multiple nutritional and lifestyle factors were associated with predicted cognitive impairment risk not only independently but also through interactions with diverse clinical and socioeconomic characteristics, collectively contributing to the model’s predictive performance.

##### 3.4.2.3 Force plot

The SHAP force plot analysis revealed distinct predictive patterns for normal cognition versus cognitive impairment (Figure 7C). For normal cognition prediction, the model assigned a 96.3% probability (substantially higher than the 43.9% baseline), with key associations coming from socioeconomic factors (education +0.121 SHAP value, PIR + 0.104) and the nutritional factor vitamin B2 (+0.0158). Moisture (+0.0645) also showed a notable association with lower predicted risk. Conversely, the cognitive impairment prediction probability was only 3.71% (below the 56.1% baseline). Notably, vitamin B2 appeared as the strongest individual factor associated with lower predicted risk, while socioeconomic variables collectively showed nearly equivalent contributions. These results illustrate how multiple protective factors were associated with lower predicted cognitive impairment risk, with vitamin B2 and socioeconomic status particularly influential in this example.

### 3.5 Cross-scale performance comparison

Optimal models: Ensemble learning methods (particularly LightGBM, XGBoost, and Random Forest) consistently showed the best performance across all assessment scales, with higher metrics compared to traditional models (e.g., SVM, K-NN) and Naive Bayes. For CERAD-WL: LightGBM achieved the highest overall performance (accuracy 0.987, AUC-ROC 0.996). For DSST: Random Forest performed slightly better (accuracy 0.877, AUC-ROC 0.94), though the differences among the three ensemble models were minimal. For AFT: Random Forest showed the strongest performance (accuracy 93.5%, AUC-ROC 0.981). The ensemble models consistently yielded AUC-ROC and AUC-PR scores above 0.95, reflecting strong discriminative ability and robustness. Naive Bayes showed the lowest performance (e.g., only 0.49 accuracy for CERAD-WL).

### 3.6 Summary of core predictors and key findings across models

To synthesize findings from our machine learning models, this section summarizes predictors associated with cognitive impairment and their interaction patterns across the CERAD-WL, DSST, and AFT assessments. SHAP analysis revealed a hierarchy of predictor importance and identified interactive patterns among variables.

#### 3.6.1 Core predictors demonstrating cross-model consistency

Sociodemographic and clinical factors showed the strongest predictive capacity. Advanced age maintained a stable association with increased risk of cognitive impairment across all models, while higher educational attainment was consistently correla ted with reduced risk. Lower family PIR was persistently associated with elevated risk scores. Among clinical indicators, hypertension and diabetes status showed stable associations with increased risk scores across all models.

After controlling for these strong predictors and applying multicollinearity filtering (VIF ≤ 3), Vitamin B2 (Riboflavin) was the only nutritional marker that demonstrated protective associations across all three cognitive domains, with higher intake levels correlating with lower model-predicted risk scores.

#### 3.6.2 Complex patterns: variable interactions and association variations

Beyond core predictors, the analysis revealed model-specific association patterns.

##### 3.6.2.1 Inter-variable interactions

SHAP analysis indicated interactive patterns among variables. A positive association was observed between vitamin B2 and vitamin E; the protective association of vitamin E was more pronounced in individuals with higher vitamin B2 levels. Similarly, the protective association of vitamin B2 was stronger under conditions of higher copper levels. In the AFT model, an interaction between zinc and iron was observed, manifesting as lower zinc being associated with higher risk when iron levels were low, while higher zinc correlated with lower risk when iron levels were high.

##### 3.6.2.2 Subgroup association differences

The strength of nutritional associations varied across population subgroups. The association between higher moisture intake and risk scores was more evident among individuals with lower education levels and those with hypertension. The protective association of vitamin B2 was more prominent in individuals with lower education. The association between caffeine intake and risk scores was more marked in low-income groups. Among different BMI groups, the protective association of vitamin D was most prominent in individuals with moderate BMI, while the association between sodium intake and risk scores was primarily observed in diabetic participants.

#### 3.6.3 Interpretation guidelines

These findings require cautious interpretation, particularly regarding nutritional factors. A primary consideration is the multicollinearity among dietary nutrients, as they typically coexist in foods. This makes it difficult to determine the independent role of any single nutrient from observational data. Therefore, while vitamin B2 demonstrates a stable cross-model signal, for other individual nutrients, the results suggest focusing on the implications of overall dietary patterns.

### 3.7 *In vitro* validation of neuroprotective nutrients identified by SHAP analysis

**Figure 8:**
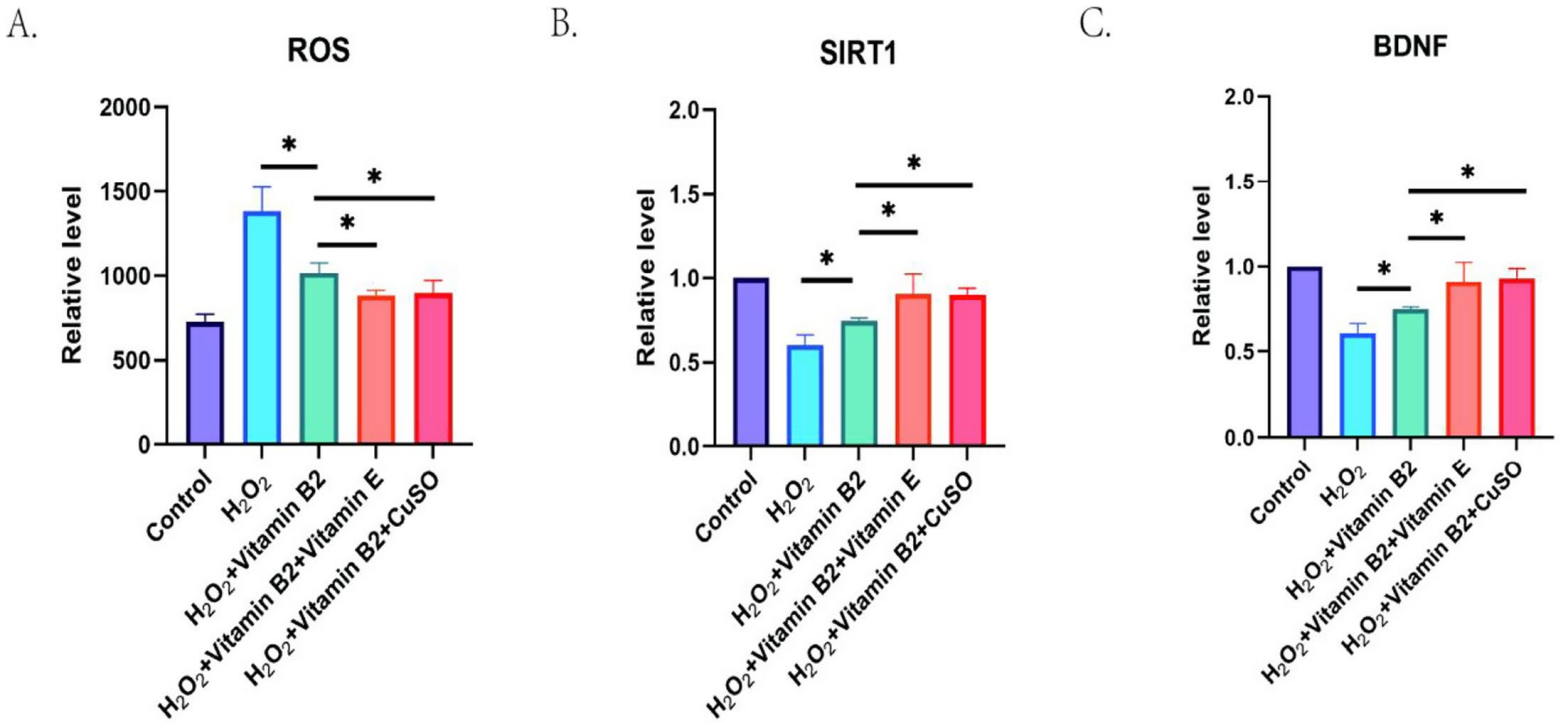
Effects of different treatments on ROS, SIRT1, and BDNF levels. **(A)** Relative level of ROS under different treatment conditions, **(B)** relative level of SIRT1 under different treatment conditions, **(C)** relative level of BDNF under different treatment conditions (*p < 0.05, **p < 0.01).

To functionally validate the nutrient associations identified through SHAP analysis, we examined whether vitamin B2 and its predicted interactions with vitamin E and copper could modulate oxidative stress and neuroprotective pathways *in vitro*. As shown in Figure 8A, treatment with H2O2 was associated with increased intracellular ROS levels, indicating successful establishment of the oxidative stress model. Pretreatment with vitamin B2 was associated with lower ROS levels. Furthermore, combined treatment with vitamin B2 and vitamin E or vitamin B2 and copper was associated with further decreases in ROS generation, consistent with a potential synergistic antioxidant pattern.

To further examine molecular responses, we measured the mRNA expression levels of SIRT1 and BDNF using qPCR. As shown in Figures 8B,C, H2O2 treatment was associated with reduced expression of SIRT1 and BDNF, whereas vitamin B2 was associated with higher expression levels compared to H2O2 alone. Combined treatment with vitamin E or copper was associated with further increases in SIRT1 and BDNF expression, suggesting enhanced upregulation under co-treatment conditions.

These findings provide experimental evidence consistent with the nutrient interactions suggested by SHAP analysis and support the biological plausibility of vitamin B2 as a factor associated with oxidative stress modulation and neurotrophic pathway activation.

## 4 Discussion

This study demonstrates the value of integrating interpretable machine learning with population-scale data to advance cognitive impairment screening. By systematically combining three neuropsychological tests with extensive NHANES data, we developed predictive models that not only achieved strong performance but also provided meaningful insights into the complex interplay between sociodemographic, clinical, and nutritional factors in cognitive health.

Established sociodemographic factors—particularly age, education level, and socioeconomic status—consistently dominated across all cognitive assessments, reinforcing their fundamental role in cognitive risk stratification (54). More importantly, our approach precisely quantified how these non-modifiable factors establish a baseline risk profile, thereby highlighting the urgent need to identify modifiable protective factors that can benefit individuals across different risk categories.

Among nutritional factors, vitamin B2 emerged as the most robust and consistent predictor, maintaining significant associations across all three cognitive domains even after rigorous adjustment for confounders and multicollinearity. This primary finding was further refined through the identification of nutrient synergy patterns, particularly between vitamin B2 and copper/vitamin E. While the individual biological pathways involved in vitamin-mediated neuroprotection are well-documented (26, 55–57), our contribution lies in demonstrating how interpretable machine learning can identify specific nutrient combinations worthy of experimental investigation. Subsequent validation experiments confirmed that these computationally predicted interactions produce measurable synergistic effects on oxidative stress reduction and neurotrophic pathway activation (12–14), thereby strengthening the biological plausibility of our population-level findings.

It is crucial to emphasize that multicollinearity among nutritional factors, comorbidities, and BMI represents an important limitation in interpreting our results. This complex covariance structure primarily stems from: (1) the inherent coexistence of dietary nutrients in natural foods; (2) socioeconomic status as a common upstream determinant influencing both dietary patterns and disease risk; and (3) the bidirectional network of associations between nutritional status, metabolic diseases (such as diabetes and hypertension), and BMI. Although we applied stringent criteria (VIF ≤ 3) to reduce statistical multicollinearity, accurately isolating the independent physiological effects of specific nutrients from these complex observational associations remains challenging.

Based on this methodological limitation, the interpretation of SHAP values for most individual nutritional factors should be approached with caution. Except for vitamin B2, which remained robust after multicollinearity control, the association results for other nutrients are better understood as emphasizing the importance of overall dietary patterns rather than providing conclusive evidence for the independent effects of any single nutrient.

The context-dependent nature of nutritional associations represents another key insight. The presence of modified effects of nutrients such as vitamin B2, caffeine, and vitamin D across different socioeconomic and metabolic subgroups further demonstrates that the impact of nutrition on cognition must be understood within the complex network of individual health status and social determinants (58–62). This understanding aligns with the concept of precision nutrition and suggests that effective dietary recommendations should consider these interacting factors.

Methodologically, our work confirms several important advantages of the integrated approach we employed. The complementary nature of the three neuropsychological tests enabled comprehensive cognitive assessment across multiple domains, while ensemble machine learning methods effectively handled the high-dimensional, nonlinear relationships inherent in these complex data (63, 64). Most significantly, SHAP analysis transformed these advanced models into interpretable tools that not only provided accurate predictions but also generated biologically plausible hypotheses about nutrient interactions—hypotheses that were subsequently validated through targeted experimentation.

We classified low cognitive performance using a ≤ 25th percentile per-test rule rather than age−/education-normed clinical cutoff (40, 41). Although this aligns with NHANES practice and facilitates population comparisons, alternative cut points (e.g., norm-referenced thresholds or z-score composites) may shift case assignments. Therefore, our findings should be interpreted as screening-oriented associations in survey data rather than clinical diagnoses; future work should compare definitions across thresholds and validate in longitudinal cohorts.

Another limitation relates to the cognitive assessments used in NHANES. The CERAD-WL, DSST, and AFT are brief screening tools designed for large-scale epidemiologic surveys rather than comprehensive neuropsychological evaluation. Although they capture key domains relevant to aging—episodic memory, processing speed/executive attention, and semantic fluency—they do not include more in-depth measures of short-term memory, long-term memory consolidation, or phonemic fluency (15–17, 65, 66). As a result, our models may not fully characterize how vitamin B2, vitamin E, or copper relate to more specific cognitive subcomponents. Future studies incorporating extended neuropsychological batteries or domain-specific tests could provide finer-grained insight into nutrient–cognition relationships and help determine whether distinct nutrient interactions differentially influence particular cognitive processes.

In addition, the cross-sectional design also limits the establishment of causal inference. Future longitudinal studies and expanded mechanistic investigations—particularly exploring pathways beyond oxidative stress, such as neuroinflammation and mitochondrial function—will be valuable next steps.

Although the ensemble models achieved very high discrimination across tasks, with AUC-ROC and AUC-PR values frequently exceeding 0.95 (and up to 0.99 in the CERAD-WL task), these values should be interpreted with caution and generalized cautiously. All reported metrics reflect internal stratified 5-fold cross-validation within NHANES under our case definition (cognitive impairment defined as ≤25th percentile on each cognitive test). This setup yields relatively clear separation between “impaired” and “non-impaired” groups and may inflate apparent model performance compared with prospective clinical screening. Even though we minimized information leakage by fitting all preprocessing steps (including standardization and class balancing) within each training fold, the present results may still overestimate real-world generalizability. External validation in independent cohorts, ideally with longitudinal cognitive outcomes, will be required to determine clinical utility.

Despite these limitations, our findings have important implications for cognitive health strategies. The strong predictive performance of models based on easily obtainable interview data supports their potential utility in community screening settings. More importantly, the identification of vitamin B2 as a robust nutritional factor that withstood multicollinearity testing, along with its synergistic partners, provides precise targets for developing stratified nutritional approaches to cognitive preservation. By successfully translating computational predictions into experimentally validated biological effects, this work demonstrates how to integrate data-driven discovery and mechanistic research to generate actionable insights for public health.

## 5 Conclusion

This study demonstrates that multidimensional predictive models based on interpretable machine learning can effectively identify populations at high risk for cognitive impairment. Our model analysis reveals the prominent value of vitamin B2 as a robust predictor across cognitive domains and identifies its synergistic patterns with copper and vitamin E. Targeting the key nutrient interactions identified by the model, *in vitro* experiments provided functional validation at the mechanistic level: combined intervention with vitamin B2 and copper/vitamin E indeed exerts synergistic effects through modulating oxidative stress levels and activating neurotrophic pathways. This “computational discovery-experimental validation” research paradigm not only strengthens the application value of vitamin B2 in the field of cognitive health but, more importantly, establishes a complete evidence chain from population data discovery to biological mechanism verification, providing new insights for developing stratified prevention and control strategies for cognitive impairment based on nutritional interventions.

## Data Availability

Publicly available datasets were analyzed in this study. This data can be found at: https://www.cdc.gov/nchs/nhanes/index.htm.
