# Supplementary material for: Interpretable machine learning models for predicting cognitive impairment using NHANES neuropsychological tests: nutritional and sociodemographic associations
Source: Front Nutr. 2026 Jan 14;12:1680290. doi: 10.3389/fnut.2025.1680290 (PMC12847056; doi:10.3389/fnut.2025.1680290)
Supplement: Supplementary file 1 [file Table_1.docx]

**Supplement 1 .Baseline characteristics of participants by CERAD-WL**

| **Characteristic** | **Overall**  N = 2,208 | **No Cognitive impairment**  N = 2,163 | **Cognitive impairment**  N = 45 | **p** |
| --- | --- | --- | --- | --- |
| Age(year)^a^, Mean ± SD | 69.23 ± 6.71 | 69.12 ± 6.68 | 74.49 ± 6.27 | <0.001 |
| Sex^b^, n(%) |  |  |  | 0.978 |
| FeMale | 1,084 (49.09%) | 1,062 (49.10%) | 22 (48.89%) |  |
| Male | 1,124 (50.91%) | 1,101 (50.90%) | 23 (51.11%) |  |
| Race/ethnicity^b^, n(%) |  |  |  | 0.553 |
| Mexican | 187 (8.47%) | 185 (8.55%) | 2 (4.44%) |  |
| Other Hispanic | 217 (9.83%) | 210 (9.71%) | 7 (15.56%) |  |
| Non-Hispanic White | 1,127 (51.04%) | 1,106 (51.13%) | 21 (46.67%) |  |
| Non-Hispanic Black | 490 (22.19%) | 478 (22.10%) | 12 (26.67%) |  |
| Other Race | 187 (8.47%) | 184 (8.51%) | 3 (6.67%) |  |
| Education^b^, n(%) |  |  |  | 0.003 |
| Less Than 9th | 221 (10.01%) | 208 (9.62%) | 13 (28.89%) |  |
| 9-11th | 278 (12.59%) | 271 (12.53%) | 7 (15.56%) |  |
| High School | 516 (23.37%) | 509 (23.53%) | 7 (15.56%) |  |
| Some College | 653 (29.57%) | 641 (29.63%) | 12 (26.67%) |  |
| College Graduate | 540 (24.46%) | 534 (24.69%) | 6 (13.33%) |  |
| Family income to poverty ratio^a^, Mean ± SD | 2.67 ± 1.61 | 2.68 ± 1.61 | 2.31 ± 1.47 | 0.120 |
| BMI^a^, Mean ± SD | 29.19 ± 6.40 | 29.23 ± 6.41 | 26.93 ± 5.40 | 0.010 |
| Smoking status^b^, n(%) |  |  |  | 0.887 |
| No | 1,929 (87.36%) | 1,890 (87.38%) | 39 (86.67%) |  |
| Yes | 279 (12.64%) | 273 (12.62%) | 6 (13.33%) |  |
| Drinking status^b^, n(%) |  |  |  | 0.464 |
| No | 354 (16.03%) | 345 (15.95%) | 9 (20.00%) |  |
| Yes | 1,854 (83.97%) | 1,818 (84.05%) | 36 (80.00%) |  |
| Diabetes ^b^, n(%) |  |  |  | 0.372 |
| No | 1693 (76.68%) | 1661 (76.79%) | 32 (71.11%) |  |
| Yes | 515 (23.32%) | 502 (23.21%) | 13 (28.89%) |  |
| Hypertension ^b^, n(%) |  |  |  | 0.058 |
| No | 839 (38.00%) | 828 (38.28%) | 11 (24.44%) |  |
| Yes | 1,369 (62.00%) | 1,335 (61.72%) | 34 (75.56%) |  |
| Energy^a^, Mean ± SD | 1,870.76 ± 790.66 | 1,873.90 ± 788.37 | 1,719.56 ± 890.21 | 0.118 |
| Protein^a^, Mean ± SD | 73.26 ± 35.91 | 73.40 ± 35.86 | 66.70 ± 38.08 | 0.090 |
| Carbohydrate^a^, Mean ± SD | 226.06 ± 99.54 | 226.14 ± 99.07 | 222.15 ± 121.34 | 0.461 |
| Total Sugar^a^, Mean ± SD | 96.36 ± 59.86 | 96.21 ± 59.61 | 103.71 ± 71.23 | 0.749 |
| Dietary fiber^a^, Mean ± SD | 17.15 ± 10.35 | 17.21 ± 10.37 | 14.28 ± 9.13 | 0.045 |
| Total Fat^a^, Mean ± SD | 72.07 ± 39.51 | 72.31 ± 39.57 | 60.77 ± 34.95 | 0.046 |
| Saturated fatty acids^a^, Mean ± SD | 22.72 ± 13.72 | 22.76 ± 13.71 | 20.89 ± 14.10 | 0.260 |
| Monounsaturated fatty acids^a^, Mean ± SD | 25.77 ± 15.17 | 25.88 ± 15.21 | 20.25 ± 11.64 | 0.012 |
| Polyunsaturated fatty acids^a^, Mean ± SD | 17.40 ± 11.32 | 17.47 ± 11.34 | 14.10 ± 10.29 | 0.016 |
| Cholesterol^a^, Mean ± SD | 268.23 ± 215.20 | 269.12 ± 215.94 | 225.67 ± 172.68 | 0.191 |
| Vitamin E as alpha-tocopherol^a^, Mean ± SD | 8.32 ± 6.62 | 8.35 ± 6.63 | 6.87 ± 5.86 | 0.018 |
| Alpha-tocopherol^a^, Mean ± SD | 0.79 ± 3.92 | 0.79 ± 3.95 | 0.62 ± 2.40 | 0.225 |
| Retinol^a^, Mean ± SD | 421.62 ± 718.21 | 420.10 ± 721.23 | 494.76 ± 555.22 | 0.613 |
| Vitamin A^a^, Mean ± SD | 657.99 ± 936.89 | 656.11 ± 941.02 | 748.09 ± 712.65 | 0.355 |
| Alpha-carotene^a^, Mean ± SD | 444.98 ± 1,807.94 | 439.58 ± 1,812.99 | 704.64 ± 1,541.03 | 0.464 |
| Beta-carotene^a^, Mean ± SD | 2,568.05 ± 6,372.00 | 2,566.73 ± 6,405.11 | 2,631.33 ± 4,553.29 | 0.836 |
| Beta-cryptoxanthin^a^, Mean ± SD | 105.26 ± 610.13 | 104.76 ± 614.21 | 129.33 ± 366.97 | 0.648 |
| Lycopene^a^, Mean ± SD | 4,479.47 ± 8,793.72 | 4,524.32 ± 8,855.62 | 2,323.24 ± 4,533.39 | 0.016 |
| Lutein+zeaxanthin^a^, Mean ± SD | 1,764.40 ± 4,054.16 | 1,772.60 ± 4,084.56 | 1,370.33 ± 2,119.31 | 0.915 |
| Thiamin(Vitamin B1) ^a^, Mean ± SD | 1.48 ± 0.74 | 1.48 ± 0.74 | 1.38 ± 0.79 | 0.193 |
| Riboflavin(Vitamin B2) ^a^, Mean ± SD | 1.96 ± 1.10 | 1.97 ± 1.10 | 1.91 ± 1.07 | 0.725 |
| Niacin^a^, Mean ± SD | 22.65 ± 12.61 | 22.70 ± 12.62 | 20.26 ± 11.70 | 0.125 |
| Vitamin B6^a^, Mean ± SD | 1.94 ± 1.33 | 1.94 ± 1.33 | 1.93 ± 1.47 | 0.444 |
| Total folate^a^, Mean ± SD | 381.07 ± 233.87 | 381.70 ± 233.15 | 350.67 ± 267.61 | 0.128 |
| Folic acid^a^, Mean ± SD | 166.34 ± 165.72 | 166.16 ± 164.27 | 174.84 ± 227.18 | 0.673 |
| Food folate^a^, Mean ± SD | 214.77 ± 145.70 | 215.58 ± 146.21 | 175.73 ± 113.52 | 0.020 |
| Folate(DFE) ^a^, Mean ± SD | 497.50 ± 332.90 | 498.01 ± 331.02 | 473.02 ± 417.15 | 0.200 |
| Total choline^a^, Mean ± SD | 314.84 ± 176.11 | 315.61 ± 176.39 | 278.24 ± 159.48 | 0.124 |
| Vitamin B12^a^, Mean ± SD | 4.74 ± 7.96 | 4.73 ± 8.02 | 4.88 ± 4.47 | 0.980 |
| Added vitamin B12^a^, Mean ± SD | 0.95 ± 2.35 | 0.94 ± 2.35 | 1.14 ± 2.54 | 0.718 |
| Vitamin C^a^, Mean ± SD | 83.23 ± 89.63 | 83.11 ± 90.02 | 88.67 ± 68.96 | 0.213 |
| Vitamin K^a^, Mean ± SD | 128.23 ± 373.60 | 128.76 ± 377.05 | 102.53 ± 121.00 | 0.146 |
| Calcium^a^, Mean ± SD | 846.94 ± 496.98 | 846.68 ± 491.35 | 859.13 ± 725.22 | 0.484 |
| Phosphorus^a^, Mean ± SD | 1,237.55 ± 566.88 | 1,238.69 ± 563.22 | 1,182.91 ± 727.15 | 0.172 |
| Magnesium^a^, Mean ± SD | 286.41 ± 137.56 | 287.28 ± 137.81 | 244.18 ± 119.28 | 0.034 |
| Iron^a^, Mean ± SD | 14.03 ± 8.12 | 14.04 ± 8.10 | 13.46 ± 9.13 | 0.339 |
| Zinc^a^, Mean ± SD | 10.05 ± 5.92 | 10.07 ± 5.93 | 9.26 ± 5.50 | 0.264 |
| Copper^a^, Mean ± SD | 1.24 ± 1.33 | 1.24 ± 1.34 | 1.00 ± 0.52 | 0.025 |
| Sodium^a^, Mean ± SD | 3,105.00 ± 1,436.38 | 3,112.60 ± 1,437.54 | 2,739.73 ± 1,344.31 | 0.055 |
| Potassium^a^, Mean ± SD | 2,575.39 ± 1,188.65 | 2,578.29 ± 1,186.66 | 2,435.96 ± 1,287.26 | 0.359 |
| Selenium^a^, Mean ± SD | 103.77 ± 60.86 | 103.98 ± 61.03 | 93.68 ± 51.45 | 0.147 |
| Caffeine^a^, Mean ± SD | 153.50 ± 177.47 | 153.94 ± 177.80 | 132.40 ± 160.74 | 0.422 |
| Theobromine^a^, Mean ± SD | 32.41 ± 79.24 | 32.24 ± 78.67 | 40.91 ± 104.01 | 0.962 |
| Alcohol^a^, Mean ± SD | 7.57 ± 21.22 | 7.60 ± 21.32 | 6.20 ± 15.88 | 0.640 |
| Moisture^a^, Mean ± SD | 2,622.60 ± 1,232.44 | 2,630.51 ± 1,233.86 | 2,242.60 ± 1,109.15 | 0.009 |
| Vitamin D (D2 + D3) ^a^, Mean ± SD | 4.60 ± 5.14 | 4.57 ± 5.11 | 5.84 ± 6.30 | 0.344 |
| a: Student t-test, b: Chi-square test, SD: standard deviation | | |  |  |

**Supplement 2 .Baseline characteristics of participants by DSST**

| **Characteristic** | **Overall**  N = 2,208 | **No Cognitive impairment**  N = 1,713 | **Cognitive impairment**  N = 495 | **p** |
| --- | --- | --- | --- | --- |
| Age(year)^a^, Mean ± SD | 69.23 ± 6.71 | 68.64 ± 6.58 | 71.26 ± 6.76 | <0.001 |
| Sex^b^, n(%) |  |  |  | <0.001 |
| FeMale | 1,084 (49.09%) | 880 (51.37%) | 204 (41.21%) |  |
| Male | 1,124 (50.91%) | 833 (48.63%) | 291 (58.79%) |  |
| Race/ethnicity^b^, n(%) |  |  |  | <0.001 |
| Mexican | 187 (8.47%) | 119 (6.95%) | 68 (13.74%) |  |
| Other Hispanic | 217 (9.83%) | 126 (7.36%) | 91 (18.38%) |  |
| Non-Hispanic White | 1,127 (51.04%) | 974 (56.86%) | 153 (30.91%) |  |
| Non-Hispanic Black | 490 (22.19%) | 325 (18.97%) | 165 (33.33%) |  |
| Other Race | 187 (8.47%) | 169 (9.87%) | 18 (3.64%) |  |
| Education^b^, n(%) |  |  |  | <0.001 |
| Less Than 9th | 221 (10.01%) | 52 (3.04%) | 169 (34.14%) |  |
| 9-11th | 278 (12.59%) | 173 (10.10%) | 105 (21.21%) |  |
| High School | 516 (23.37%) | 400 (23.35%) | 116 (23.43%) |  |
| Some College | 653 (29.57%) | 577 (33.68%) | 76 (15.35%) |  |
| College Graduate | 540 (24.46%) | 511 (29.83%) | 29 (5.86%) |  |
| Family income to poverty ratio^a^, Mean ± SD | 2.67 ± 1.61 | 2.94 ± 1.60 | 1.72 ± 1.25 | <0.001 |
| BMI^a^, Mean ± SD | 29.19 ± 6.40 | 29.22 ± 6.41 | 29.06 ± 6.39 | 0.595 |
| Smoking status^b^, n(%) |  |  |  | <0.001 |
| No | 1,929 (87.36%) | 1,520 (88.73%) | 409 (82.63%) |  |
| Yes | 279 (12.64%) | 193 (11.27%) | 86 (17.37%) |  |
| Drinking status^b^, n(%) |  |  |  | <0.001 |
| No | 354 (16.03%) | 244 (14.24%) | 110 (22.22%) |  |
| Yes | 1,854 (83.97%) | 1,469 (85.76%) | 385 (77.78%) |  |
| Diabetes |  |  |  | <0.001 |
| No | 1693 (76.68%) | 1364 (79.63%) | 329 (66.46%) |  |
| Yes | 515 (23.32%) | 349 (20.37%) | 166 (33.54%) |  |
| Hypertension ^b^, n(%) |  |  |  | <0.001 |
| No | 839 (38.00%) | 695 (40.57%) | 144 (29.09%) |  |
| Yes | 1,369 (62.00%) | 1,018 (59.43%) | 351 (70.91%) |  |
| Energy^a^, Mean ± SD | 1,870.76 ± 790.66 | 1,925.80 ± 783.56 | 1,680.28 ± 786.29 | <0.001 |
| Protein^a^, Mean ± SD | 73.26 ± 35.91 | 74.74 ± 33.99 | 68.15 ± 41.54 | <0.001 |
| Carbohydrate^a^, Mean ± SD | 226.06 ± 99.54 | 231.01 ± 99.23 | 208.91 ± 98.82 | <0.001 |
| Total Sugar^a^, Mean ± SD | 96.36 ± 59.86 | 98.86 ± 59.93 | 87.74 ± 58.87 | <0.001 |
| Dietary fiber^a^, Mean ± SD | 17.15 ± 10.35 | 17.60 ± 10.34 | 15.62 ± 10.26 | <0.001 |
| Total Fat^a^, Mean ± SD | 72.07 ± 39.51 | 74.99 ± 39.81 | 61.97 ± 36.71 | <0.001 |
| Saturated fatty acids^a^, Mean ± SD | 22.72 ± 13.72 | 23.63 ± 13.85 | 19.60 ± 12.78 | <0.001 |
| Monounsaturated fatty acids^a^, Mean ± SD | 25.77 ± 15.17 | 26.76 ± 15.30 | 22.33 ± 14.17 | <0.001 |
| Polyunsaturated fatty acids^a^, Mean ± SD | 17.40 ± 11.32 | 18.18 ± 11.62 | 14.70 ± 9.77 | <0.001 |
| Cholesterol^a^, Mean ± SD | 268.23 ± 215.20 | 273.08 ± 213.46 | 251.46 ± 220.51 | 0.002 |
| Vitamin E as alpha-tocopherol^a^, Mean ± SD | 8.32 ± 6.62 | 8.72 ± 6.83 | 6.91 ± 5.61 | <0.001 |
| Alpha-tocopherol^a^, Mean ± SD | 0.79 ± 3.92 | 0.82 ± 4.11 | 0.66 ± 3.19 | 0.854 |
| Retinol^a^, Mean ± SD | 421.62 ± 718.21 | 439.16 ± 776.99 | 360.94 ± 455.58 | <0.001 |
| Vitamin A^a^, Mean ± SD | 657.99 ± 936.89 | 686.89 ± 1,008.88 | 557.96 ± 617.40 | <0.001 |
| Alpha-carotene^a^, Mean ± SD | 444.98 ± 1,807.94 | 469.94 ± 1,981.69 | 358.60 ± 991.88 | 0.011 |
| Beta-carotene^a^, Mean ± SD | 2,568.05 ± 6,372.00 | 2,698.66 ± 6,807.98 | 2,116.05 ± 4,528.46 | <0.001 |
| Beta-cryptoxanthin^a^, Mean ± SD | 105.26 ± 610.13 | 92.40 ± 248.52 | 149.75 ± 1,202.70 | 0.021 |
| Lycopene^a^, Mean ± SD | 4,479.47 ± 8,793.72 | 4,695.76 ± 9,183.32 | 3,730.97 ± 7,245.01 | 0.007 |
| Lutein+zeaxanthin^a^, Mean ± SD | 1,764.40 ± 4,054.16 | 1,879.11 ± 4,342.07 | 1,367.44 ± 2,808.61 | <0.001 |
| Thiamin(Vitamin B1) ^a^, Mean ± SD | 1.48 ± 0.74 | 1.52 ± 0.74 | 1.34 ± 0.73 | <0.001 |
| Riboflavin(Vitamin B2) ^a^, Mean ± SD | 1.96 ± 1.10 | 2.03 ± 1.11 | 1.74 ± 1.01 | <0.001 |
| Niacin^a^, Mean ± SD | 22.65 ± 12.61 | 23.23 ± 12.49 | 20.65 ± 12.80 | <0.001 |
| Vitamin B6^a^, Mean ± SD | 1.94 ± 1.33 | 1.98 ± 1.33 | 1.79 ± 1.33 | <0.001 |
| Total folate^a^, Mean ± SD | 381.07 ± 233.87 | 395.54 ± 239.23 | 330.98 ± 206.85 | <0.001 |
| Folic acid^a^, Mean ± SD | 166.34 ± 165.72 | 172.30 ± 169.86 | 145.71 ± 148.83 | <0.001 |
| Food folate^a^, Mean ± SD | 214.77 ± 145.70 | 223.30 ± 151.07 | 185.25 ± 120.95 | <0.001 |
| Folate(DFE) ^a^, Mean ± SD | 497.50 ± 332.90 | 516.12 ± 340.08 | 433.07 ± 298.22 | <0.001 |
| Total choline^a^, Mean ± SD | 314.84 ± 176.11 | 320.16 ± 171.17 | 296.44 ± 191.25 | <0.001 |
| Vitamin B12^a^, Mean ± SD | 4.74 ± 7.96 | 4.87 ± 8.56 | 4.27 ± 5.33 | <0.001 |
| Added vitamin B12^a^, Mean ± SD | 0.95 ± 2.35 | 1.01 ± 2.44 | 0.74 ± 2.02 | <0.001 |
| Vitamin C^a^, Mean ± SD | 83.23 ± 89.63 | 85.57 ± 91.91 | 75.13 ± 80.84 | 0.003 |
| Vitamin K^a^, Mean ± SD | 128.23 ± 373.60 | 138.29 ± 416.62 | 93.43 ± 143.13 | <0.001 |
| Calcium^a^, Mean ± SD | 846.94 ± 496.98 | 867.15 ± 494.75 | 776.99 ± 498.86 | <0.001 |
| Phosphorus^a^, Mean ± SD | 1,237.55 ± 566.88 | 1,271.40 ± 554.94 | 1,120.43 ± 592.21 | <0.001 |
| Magnesium^a^, Mean ± SD | 286.41 ± 137.56 | 295.77 ± 138.87 | 254.01 ± 127.87 | <0.001 |
| Iron^a^, Mean ± SD | 14.03 ± 8.12 | 14.35 ± 8.14 | 12.91 ± 7.96 | <0.001 |
| Zinc^a^, Mean ± SD | 10.05 ± 5.92 | 10.31 ± 5.73 | 9.16 ± 6.46 | <0.001 |
| Copper^a^, Mean ± SD | 1.24 ± 1.33 | 1.28 ± 1.44 | 1.09 ± 0.85 | <0.001 |
| Sodium^a^, Mean ± SD | 3,105.00 ± 1,436.38 | 3,189.39 ± 1,420.07 | 2,812.97 ± 1,455.55 | <0.001 |
| Potassium^a^, Mean ± SD | 2,575.39 ± 1,188.65 | 2,648.28 ± 1,182.99 | 2,323.15 ± 1,174.76 | <0.001 |
| Selenium^a^, Mean ± SD | 103.77 ± 60.86 | 105.66 ± 58.05 | 97.23 ± 69.38 | <0.001 |
| Caffeine^a^, Mean ± SD | 153.50 ± 177.47 | 162.82 ± 179.00 | 121.22 ± 168.28 | <0.001 |
| Theobromine^a^, Mean ± SD | 32.41 ± 79.24 | 34.46 ± 83.54 | 25.34 ± 61.69 | <0.001 |
| Alcohol^a^, Mean ± SD | 7.57 ± 21.22 | 8.27 ± 21.46 | 5.13 ± 20.20 | <0.001 |
| Moisture^a^, Mean ± SD | 2,622.60 ± 1,232.44 | 2,720.35 ± 1,232.37 | 2,284.32 ± 1,172.49 | <0.001 |
| Vitamin D (D2 + D3) ^a^, Mean ± SD | 4.60 ± 5.14 | 4.65 ± 5.27 | 4.42 ± 4.66 | 0.645 |
| a: Student t-test, b: Chi-square test, SD: standard deviation | | |  |  |
